# Peripheral nerve remodeling in the tumor microenvironment: neuro-immune crosstalk, molecular mechanisms, and precision therapeutic

**DOI:** 10.3389/fimmu.2026.1789297

**Published:** 2026-05-13

**Authors:** Huiying Liu, Xunjun Li, Jiang Yu, Tao Chen

**Affiliations:** 1 Department of General Surgery, Nanfang Hospital, Southern Medical University, Guangzhou, Guangdong, China; 2 Guangdong Provincial Key Laboratory of Precision Medicine for Gastrointestinal Tumor, Guangzhou, Guangdong, China; 3 Department of Gastrointestinal and Hernia Surgery, Ganzhou Hospital-Nanfang Hospital, Southern Medical University, Ganzhou, Jiangxi, China

**Keywords:** autonomic nerve differentiation, cancer therapeutic strategies, neurotrophin functional reprogramming, peripheral nervous system remodeling, tumor microenvironment, tumor-neural interactions

## Abstract

As a crucial component of the tumor microenvironment, the nervous system modulates tumor initiation, progression, and metastasis. Conversely, tumors actively hijack and reshape neural structures to activate nerve-dependent developmental and regenerative processes that promote their own growth and survival. Despite extensive progress in tumor-neural crosstalk, the molecular mechanisms underlying neuro-immune coordination in peripheral nerve remodeling remain incompletely understood, and integrated strategies targeting this axis to overcome immunotherapy resistance are still lacking. This review comprehensively examines the phenotypic and functional remodeling of the peripheral nervous system within the tumor microenvironment, with a focus on neuro-immune crosstalk between neural cells, immune cells and cancer cells. We systematically analyze phenotypic remodeling in neurons and glial cells, with particular emphasis on autonomic nerve differentiation, functional reprogramming of the neurotrophin family, and the regulatory roles of immune cells in these processes. Finally, we discuss precision therapeutic strategies including β-adrenergic blockades, neurotrophin signaling inhibition, surgical and pharmacological denervation, and combinatorial regimens with immunotherapy, highlighting their translational potential in cancer treatment.

Neuro-immune crosstalk in the tumor microenvironment: schematic of four core interaction nodes: This schematic illustrates the bidirectional interactions between peripheral nerves and immune cells within the tumor microenvironment. Schwann cells engage in antigen presentation and cytokine signaling with tumor-associated macrophages and lymphocytes. Sympathetic-derived norepinephrine drives T cell exhaustion and myeloid-derived suppressor cell expansion, while parasympathetic-derived acetylcholine activates α7 nicotinic acetylcholine receptors on immune cells to suppress NF-κB signaling. Macrophage- and lymphocyte-derived NGF and BDNF establish positive feedforward loops that amplify tumor-neural-immune interactions. Together, these interconnected mechanisms support tumor innervation, progression and immune evasion, positioning the neuro-immune axis as an emerging therapeutic target for cancer treatment.

## Graphical Abstract

**Figure:**
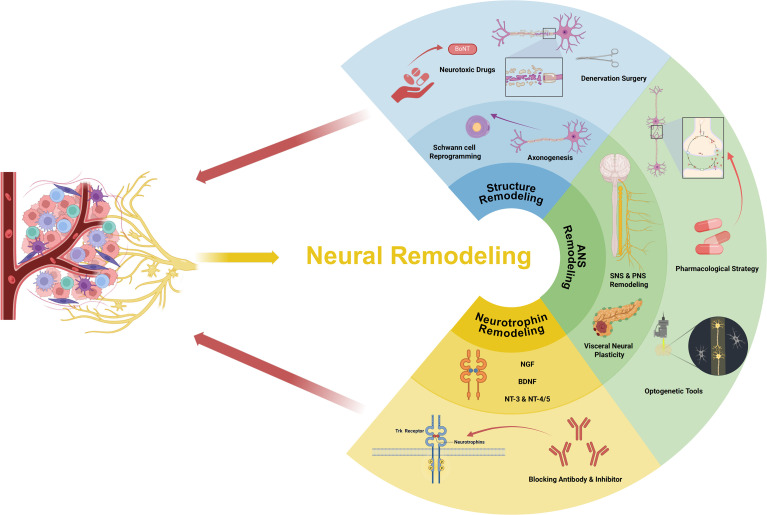
The phenotypic and mechanistic aspects of neural remodeling in the tumor microenvironment encompass the following key components: Structure remodeling, automatic nervous system(ANS) remodeling and neurotrophin remodeling. Structural alterations in neurons are manifested through tumor microenvironment-induced axonal regeneration, as well as the dedifferentiation of glial cells, particularly Schwann cells. Additionally, autonomic nerves—including both sympathetic and parasympathetic nerves—undergo differentiation and reinnervation. Concurrently, the neurotrophin family, including NGF, BDNF, NT-3, and NT-4(5), is also remodeled under the influence of various components within the tumor microenvironment. The inducible remodeling of nerves suggests novel strategies for tumor therapy, opening up new avenues for comprehensive cancer treatment through various combinations with existing modalities such as chemotherapy and immunotherapy.

## 1 Background

Tumors should not be viewed as homogeneous model structures but rather as heterogeneous ecosystems shaped by dynamic crosstalk between cancer cells and their surrounding tumor microenvironment (TME). The TME comprises a complex network of cellular and non-cellular components, including stromal cells, immune cells, blood and lymphatic vessels, extracellular matrix (ECM), and soluble factors, among which the nervous system serves as a core regulatory element. The nervous system governs organ development, regeneration, and homeostasis throughout life (1, 2), and accumulating evidence from preclinical mouse models and clinical studies of solid tumors has demonstrated that neural activity modulates tumor initiation, progression, and metastasis (2, 3).

In turn, the TME can actively remodel and hijack the structure and function of the nervous system to foster malignant progression (4, 5). Given that over 95% of malignant tumors originate outside the central nervous system (CNS) (6), this review focuses on the phenotypic and functional remodeling of the peripheral nervous system (PNS) in the TME, aiming to clarify the understudied neuro-immune crosstalk mechanisms and refine the tumor-neural interaction network by defining the cellular and molecular basis of neural remodeling. Current understanding remains limited regarding tissue-specific neural plasticity, the precise immune cell regulatory circuits, and the functional consequences of neurotrophin reprogramming in different cancer types.

**Figure 1:**
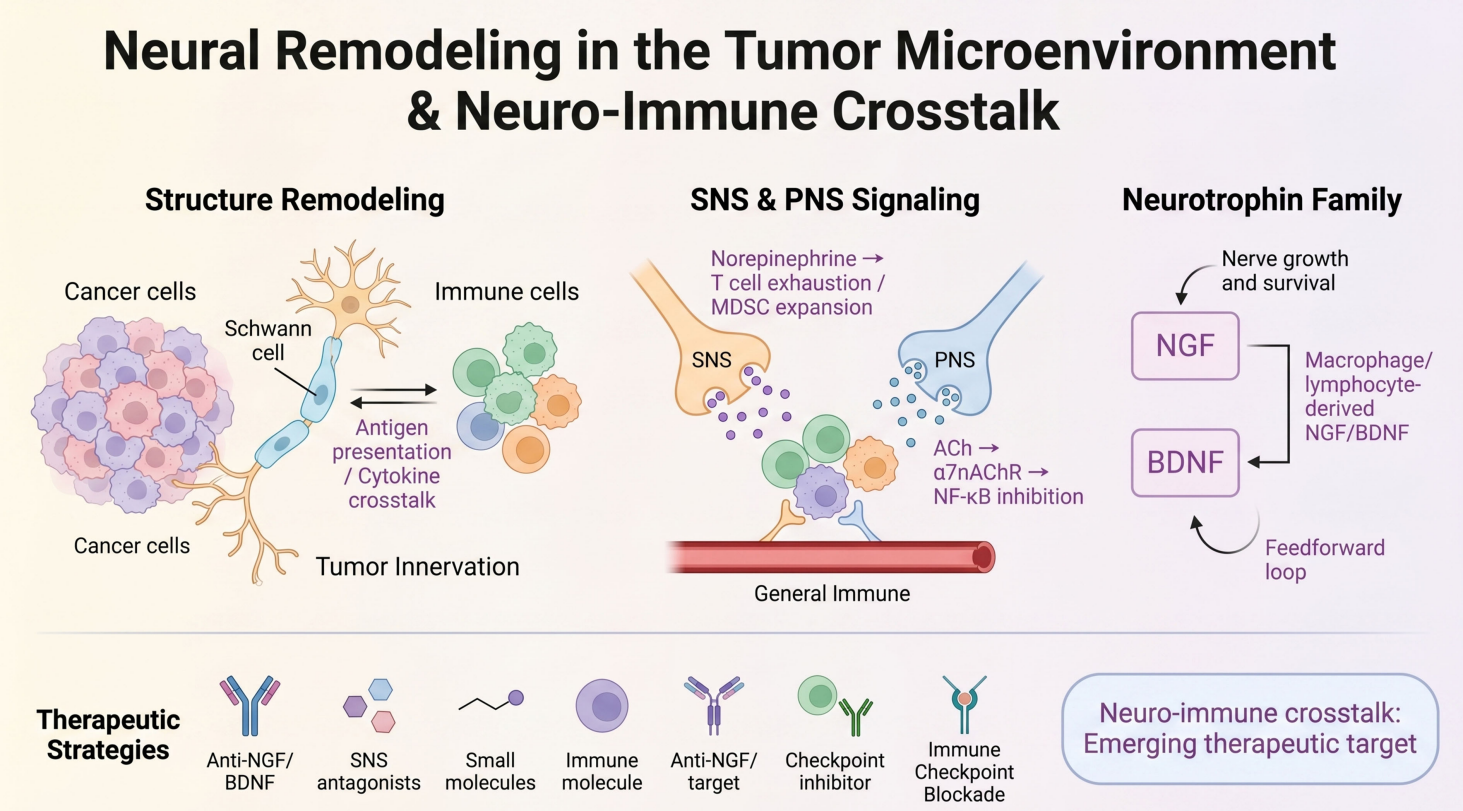
Neuro-immune crosstalk in the tumor microenvironment: schematic of four core interaction nodes: This schematic illustrates the bidirectional interactions between peripheral nerves and immune cells within the tumor microenvironment. Schwann cells engage in antigen presentation and cytokine signaling with tumor-associated macrophages and lymphocytes. Sympathetic-derived norepinephrine drives T cell exhaustion and myeloid-derived suppressor cell expansion, while parasympathetic-derived acetylcholine activates α7 nicotinic acetylcholine receptors on immune cells to suppress NF-κB signaling. Macrophage- and lymphocyte-derived NGF and BDNF establish positive feedforward loops that amplify tumor-neural-immune interactions. Together, these interconnected mechanisms support tumor innervation, progression and immune evasion, positioning the neuro-immune axis as an emerging therapeutic target for cancer treatment.

This review presents a novel perspective on the dynamic reciprocity of tumor-neural-immune interactions, as illustrated in **Figure 1** detailing structural and functional changes in the PNS, including axonogenesis, glial cell reprogramming, autonomic nerve differentiation, and neurotrophin functional remodeling. Finally, we summarize and discuss integrated therapeutic strategies targeting PNS remodeling for precision cancer intervention.

## 2 Structural remodeling of PNS in the TME

In addition to the lymphatic and vascular networks, the nervous system represents one of the primary pathways for cancer cell migration and invasion. Metastatic spread along nerve fibers is defined as perineural invasion (PNI). Histopathologically, PNI is commonly diagnosed when tumor cells surround at least 33% of the nerve circumference (7–9), a criterion used to quantify tumor-nerve contact in clinical and experimental specimens.

At the structural level, peripheral nerve fibers consist of axons and the glial cells that ensheathe them. Structural remodeling of peripheral nerves in the TME occurs through direct interactions with axons and indirect crosstalk with glial cells.

### 2.1 Axonogenesis

Axonogenesis refers to the formation and outgrowth of new axons, a process that is robustly induced in the tumor microenvironment. Since the initial discovery of nerve fibers sprouting in prostate cancer (10), extensive research has confirmed that increased nerve density is highly correlated with tumor progression. This association has been clearly established in cancers such as head and neck, breast, lung, gastric, pancreatic, prostate, colon, and rectal cancers (3, 5, 10–15). Furthermore, during tumorigenesis, nerve density exhibits a stage-specific increase alongside the progression of precancerous lesions (5, 16, 17). These newly formed nerves can release growth factors and neurotransmitters in the TME, thereby promoting tumor cell growth and stimulating the activity of tumor-supporting cells (4, 5).

Multiple mechanisms underlie enhanced axonogenesis in the TME. Tumor cells and stromal components secrete factors that promote axonal outgrowth and recruit nerve fibers, increasing local innervation. Key mediators include neurotrophins and neurotransmitters. In addition, the migration and differentiation of neural progenitor cells contribute substantially to TME−associated axonogenesis (18).

The neurotrophin family comprises several key members, including nerve growth factor (NGF) and brain−derived neurotrophic factor (BDNF), both of which strongly regulate axonogenesis. Among these, NGF is the best−characterized driver of axonogenesis in solid tumors (19, 20). In breast cancer, approximately one-third of invasive ductal carcinomas exhibit detectable nerves, with nerve density strongly correlating with NGF secretion by tumor cells (13, 21). Beyond tumor cells, other TME components, such as cancer-associated fibroblasts (CAFs), can also secrete NGF to support nerve growth and remodeling (22). Furthermore, pancreatic cancer cells recruit axons via NGF-mediated mechanisms, and the newly formed axons further promote metabolism by upregulating serine biosynthesis pathways (23), revealing the metabolic crosstalk in tumor-neural interactions. Other neurotrophins can also trigger axonogenesis through activation of downstream signaling pathways like PI3K and Ras-Raf-MAPK (24). In ovarian cancer, tumor cell–derived BDNF has been shown to activate its high−affinity receptor TrkB on sympathetic neurons, thereby promoting directional axonal outgrowth and adrenergic nerve infiltration into tumor tissues (25).

The axonogenesis process mediated by neurotrophins in the TME is often initiated by increased neurotransmitter secretion, establishing a feedforward mechanism (19). In gastric and colorectal cancer studies, tumor cells have been shown to synthesize and secrete acetylcholine as measured by colorimetric assay and high-performance liquid chromatography (HPLC) (26). Tumor-derived acetylcholine promotes axonal outgrowth (19, 26–30), as validated by neurite extension assays in primary dorsal root ganglion (DRG) cultures and ex vivo nerve explant models (19). Likewise, norepinephrine enhances bidirectional tumor-neural crosstalk by binding to β3-adrenergic receptors, upregulating BDNF expression, and stimulating axonogenesis via TrkB receptors on neural cells (25, 31–33).

Evidence of neural progenitor cell migration further clarifies and refines the mechanisms of axonogenesis in the TME. In animal models of prostate cancer, the number of neural progenitor cells expressing doublecortin (DCX) increases (18). DCX marks migratory and differentiating neural progenitor cells, and their accumulation in the TME enables these cells to invade tumor tissues via the circulation, differentiate into mature neurons, and increase intratumoral nerve density, thereby establishing functional tumor−neural crosstalk to support cancer progression. It has been demonstrated that these progenitor cells can invade prostate tumors via the bloodstream, and then differentiate into neurons (18). The TME responds to neural progenitor cell migration by upregulating extracellular matrix (ECM) proteins, including collagen, laminin, and fibronectin. Among these components, upregulated type I collagen, as a superpolymer for angiogenesis, facilitates the migration of new blood vessels and nerves (34–38). Conversely, the same dense ECM components, particularly type I collagen and hyaluronan, can act as a physical barrier that limits the intratumoral penetration of systemically administered neuroactive drugs, posing a significant translational challenge for targeting tumor-associated nerves (39).

During normal neural development and repair, the axonal growth and extension are primarily mediated by axonal guidance molecules such as ROBO-Slit or Semaphorin (40). ROBO proteins act as receptors, whereas SLIT proteins function as secreted ligands. The upregulation of these molecules in the TME (41, 42) and their role in promoting tumor cell migration and invasion are increasingly understood (43, 44), particularly in pancreatic cancer and colorectal cancer (41, 45, 46). These molecules induce cytoskeletal reorganization to facilitate metastasis (46) and modulate metabolic pathways to support tumor growth (47). Relevant studies show that tumor cells can release exosomes containing the axonal guidance molecule EphrinB1 to induce nerve growth (48). However, the specific mechanisms by which these molecules drive axonogenesis in the TME require further exploration.

### 2.2 Schwann cell reprogramming in the tumor microenvironment

Schwann cells, the most common glial cell type in the PNS (49), exhibit diverse functions, including key roles in neural development, myelination, tissue repair, and antigen presentation under inflammatory conditions (50–53). In tumor–neural crosstalk, Schwann cells exert prominent pro−tumor functions, including promotion of cancer cell invasion, perineural invasion, and poor clinical outcomes, across multiple cancer types, including pancreatic, colorectal, and thyroid cancers (54–57). Notably, tumors exploit the innate repair and regenerative properties of Schwann cells by upregulating molecular signals, activating core signaling pathways, and enhancing intercellular crosstalk within the TME, thereby inducing pro−tumor Schwann cell reprogramming (58).

During the PNS development, Schwann cells embed between axons, initiating myelination (59). Similarly, in the TME, Schwann cells interact closely with cancer cells, inducing membrane protrusions at contact sites and directing cancer cell migration toward Schwann cell–rich regions, thereby facilitating tumor invasion (54). In the TME, tumor cells induce pro-tumor phenotypic and functional alterations in Schwann cells through diverse molecular mediators, including NGF, neurotrophin−3 (NT−3), and tumor cell−derived extracellular vesicles (EVs). Pancreatic cancer cells secrete NGF to activate autophagy in Schwann cells, promoting their proliferation and migration (60). Conversely, knocking out p75 neurotrophin receptor (p75NTR) in Schwann cells or using small-molecule inhibitors to block NGF-p75NTR binding reduces Schwann cell migration toward cancer cells (55). In human pancreatic cancer and precancerous inflammatory specimens, elevated expression levels of neurotrophin−3 (NT−3) and artemin (ARTN) with its receptor GFRα3 have been observed in Schwann cells (61, 62). In pancreatic ductal adenocarcinoma (PDAC), downregulation of the axonal guidance molecule SLIT2 promotes Schwann cell migration toward cancer cells, enhancing mutual chemotaxis between nerves and cancer cells (45).

Following peripheral nerve injury, Schwann cells adopt a repair phenotype to support the regeneration and guidance of axons (63). This reprogramming involves extensive changes in gene expression, including myelination transcription factors, structural proteins, and membrane glycoproteins. Schwann cells in the TME exhibit a similar reprogramming phenotype. Consistent with the re-expression of myelination genes in Schwann cells post-injury (64), in human pancreatic, colon, thyroid, and salivary gland cancer specimens, upregulated glial fibrillary acidic protein (GFAP) expression in Schwann cells acts as a marker of reactive glial phenotype, which correlates with enhanced Schwann cell activation, migration, and pro−tumor paracrine signaling to accelerate cancer cell invasion and perineural invasion (54, 55). Digital spatial profiling reveals elevated transcriptomic signals associated with nerve injury in tumor-associated nerves in PDAC (65). Further studies confirm that this reprogramming is driven by the activation of c-Jun (66), a transcription factor that is robustly induced and activated in Schwann cells following peripheral nerve injury. Compared to adjacent normal tissues, both PDAC animal models and patient specimens show significantly increased phosphorylated c−Jun (p−cJun) expression in Schwann cells (66). Phosphorylation stabilizes c−Jun and triggers its transcriptional activity, which governs the conversion of mature Schwann cells into a repair−like phenotype, thereby promoting nerve remodeling, Schwann cell motility, and tumor progression. Highly aggressive and poorly prognostic melanomas and small cell lung cancers also exhibit Schwann cell reprogramming from mature to repair-like phenotypes (67, 68).To bridge the gap between nerve stumps after injury, reprogrammed Schwann cells undergo morphological changes to form Büngner bands and guide the axon regeneration (69). Similarly, in the TME, activated Schwann cells form dynamic guidance tracks called tumor-activated Schwann cell tracks (TAST), which physically arrange to promote tumor cell migration and invasion (66).

Interactions between Schwann cells and other TME components, such as CAFs, also induce pro-tumor functional reprogramming. CAFs secrete the ligand SLIT2, activating the SLIT2/ROBO2 pathway in Schwann cells. This signaling cascade regulates β-catenin and cadherin-2 pathways, promoting Schwann cell hyperproliferation beyond normal physiological levels, as well as migration through N-cadherin/β-catenin signaling (70). Additionally, leukemia inhibitory factor (LIF), a pleiotropic cytokine implicated in inflammation, cell differentiation, and tumor progression, derived from CAFs activates the JAK/STAT3/Akt pathway in Schwann cells, further enhancing their migratory and differentiation capacities (71). Conversely, Schwann cells mediate IL-1α signaling to drive CAF transformation into a more malignant inflammatory subtype, further inducing epithelial-mesenchymal transition (EMT) and increasing pancreatic cancer cell invasion and migration (72, 73).

Schwann cells and tumor cells also interact via extracellular vesicles, adding to the dynamism and complexity in the TME. EVs mediate critical intercellular communication in the TME by transferring proteins, lipids, and nucleic acids to regulate cell phenotype, signaling, and tumor progression. Schwann cell−derived EVs (SC−EVs) can be directly hijacked by cancer cells to enhance proliferation, survival, and invasiveness (74). Upregulation of these pro−inflammatory and pro-tumor cytokines amplifies local inflammation, enhances cancer cell proliferation and migration, promotes ECM degradation, facilitates immune suppression, and ultimately accelerates tumor invasion and metastasis. Tumor cell−derived EVs induce Schwann cell activation through IL−8 signaling, leading to NF−κB p65 nuclear translocation and triggering the production of cytokines such as IL−8, IL−1β, TNFα, IL−6, CCL2, and MMP−2 (75). In the colon TME, tumor cell-derived exosomes containing miR-21-5p upregulate NGF transcription in Schwann cells, promoting tumor cell proliferation and migration while feedback-enhancing exosomal miR-21-5p expression (74). Studies also show that tumor cells can directly hijack SC-EVs to further promote tumor cell proliferation (76).

## 2 Remodeling of autonomic nerve differentiation in the TME

Anatomically, the PNS is divided into autonomic and somatic nerves. The autonomic nervous system consists of sympathetic and parasympathetic branches (77), which jointly regulate internal homeostasis. The somatic nervous system includes sensory and somatosensory components, anatomically composed of sensory, somatic, and mixed nerves. At the neuronal level, autonomic nerves are composed of two neurons: preganglionic and postganglionic neurons (77). Preganglionic neurons originate in the spinal cord or brainstem, while postganglionic neurons are located in the PNS near the glands and organs they innervate. Thus, autonomic nerves can rapidly respond to TME changes and are more susceptible to remodeling by microenvironmental influences (18).

Notably, because autonomic neuronal cell bodies reside outside the organs they innervate, particularly in breast, ovarian, pancreatic, and gastric cancers, their genetic material may be overlooked in conventional tumor analyses, potentially underestimating their plasticity in the TME. Moreover, in the TME, peripheral nerve subpopulations exert tissue-specific effects, necessitating careful identification of the contributions of various neurotransmitters from different subpopulations. For example, in breast and ovarian cancers, sympathetic-derived β-adrenergic signaling accelerates cancer progression (78, 79), whereas parasympathetic nerves oppose this effect and inhibit cancer progression. Similarly, in pancreatic cancer, cholinergic signaling inhibits growth (80), while adrenergic signaling promotes it (81). In gastric cancer models, vagal innervation’s pro-tumor effect via cholinergic-mediated Wnt signaling is well-established, and vagotomy is considered a viable strategy to impede gastric cancer progression (3). The divergent phenotypes of peripheral nerves in different TMEs suggest tissue-specific neural remodeling, the mechanisms of which require further study.

### 2.1 Remodeling of the sympathetic nervous system in the TME

In normal physiological homeostasis, the sympathetic nervous system (SNS) regulates nearly all organ systems by locally releasing catecholamines and adrenergic neurotransmitters (82). Beyond maintaining homeostasis, SNS activation leads to local and systemic increases in adrenergic neurotransmitters, which have been shown to influence tumor growth and metastasis (83).

Sympathetic innervation is commonly observed in various peripheral cancers (i.e., solid malignancies originating outside the central nervous system) and is typically associated with poor prognosis, including breast, colorectal, pancreatic, and prostate cancers (10, 78, 84, 85). In the TME, the SNS promotes tumor progression through multiple mechanisms. For instance, β-adrenergic signaling activates classical Src signaling, inducing invasive pseudopod formation in cancer cells and enhancing breast tumor aggressiveness (86). Additionally, β-adrenergic signaling remodels intratumoral and peritumoral vascular and lymphatic networks, facilitating metastatic spread (78, 87). Further studies confirm that β-adrenergic signaling alters immune and non-immune stromal cell functions, promoting angiogenesis and T-cell exhaustion in the TME (16, 88). SNS activation may also upregulate trophic factors and chemokines (e.g., CXCL2), attracting tumor cells to nerve fibers (5, 89–91).

Conversely, TME components interact with sympathetic nerves via various mechanisms to promote neural remodeling, including mediating sympathetic nerve growth, neuronal differentiation toward sympathetic phenotypes, and visceral sympathetic reinnervation. Cells implicated in sympathetic remodeling in the TME include CAFs, lymphocytes, macrophages, and cold-induced neuroimmune cells (CINCs). These cells primarily secrete cytokines, neurotrophins, and axon guidance molecules to induce neural remodeling in sympathetic nerve fibers.

Sympathetic fibers can infiltrate peritumoral and intratumoral regions from healthy tissue (5, 10), similarly in response to neurotrophins in the TME (91). In ovarian cancer, BDNF secretion drives adrenergic nerve infiltration (25). In colorectal TME, bidirectional interactions exist between sympathetic nerves and NGF-expressing CAFs. β2-adrenergic stimulation induces CAFs to secrete NGF, expanding sympathetic innervation and further increasing norepinephrine accumulation in the TME. Elevated norepinephrine promotes tumor growth directly via ADRA2A/Gi-LATS-YAP pathway activation or indirectly by feedback-inducing NGF secretion (84). Activation of this axis suppresses Hippo signaling, promoting YAP-dependent transcription of cell cycle regulators and anti-apoptotic factors in cancer cells. These interactions make neurotrophin signaling blockade a potential therapeutic strategy for colorectal cancer (84). Similarly, in breast cancer models, particularly triple-negative breast cancer, NGF exhibits feedback upregulation of adrenergic signaling (92), highlighting the universality of sympathetic-neurotrophin interactions in the TME. Endothelial NGF receptors further promote neurovascular alignment and sympathetic sprouting in thermogenic adipose tissue, which is significant for tumor metabolism (93).

CINCs in the TME secrete BDNF, supporting sympathetic process survival and growth. CINCs can also induce phenotypic shifts in sympathetic-associated macrophages (SAMs), counteracting sympathetic reduction in the TME (94). RNA sequencing indicates that CINCs regulate synaptogenesis and neuronal branching genes (94).

Lymphocytes modulate nerve growth and organ innervation by secreting cytokines and neurotrophins. Lymphocyte-derived granulocyte-macrophage colony-stimulating factor (GM-CSF) stimulates sympathetic growth (95). This hematopoietic progenitor mobilizer has been shown to promote adrenergic innervation and prostate cancer progression (95). Additionally, co-culture experiments using isolated T cells as the predominant lymphocyte population demonstrate that T cells can mediate neuronal growth via NGF secretion (96) and differentially regulate neurotransmitter gene expression in sympathetic ganglia (97). Cytokine-neurotrophin cross-talk further modulates sympathetic growth. For example, IL-1 mediates NGF production in lymphoid tissues, inducing sympathetic growth (98).

In the TME, sympathetic neurotransmitters regulate macrophage cytokine expression (99), and macrophages exhibit potential to remodel sympathetic growth and differentiation. Dynamic interactions between adipose tissue macrophages and sympathetic fibers suggest macrophages may promote sympathetic plasticity in the TME (94). M2-like macrophages, known for their pro-tumor and immunosuppressive functions, secrete SLIT3. This ligand binds to ROBO1 receptors on sympathetic neurons, activating protein kinase A (PKA) signaling to increase norepinephrine release and feedback-promote sympathetic growth (100). SAMs in the TME uptake and degrade norepinephrine, modulating local adrenergic signaling (101). Macrophages also mediate angiogenesis, enhance Schwann cell motility, remodel ECM, and interact with endothelial cells (102), underscoring the role as key influencers of sympathetic differentiation in the TME.

Beyond TME-mediated sympathetic fiber growth, direct evidence shows the TME regulates neuronal differentiation toward sympathetic phenotypes. Experiments reveal that TP53-deficient head and neck tumor cells transmit signals via exosomes to adjacent neurons, causing adrenergic phenotypic reprogramming of sensory nerves in the TME. This alters neuronal function and axon sprouting to promote tumor growth (103), suggesting diverse modes of tumor-sympathetic crosstalk that require systematic mechanistic studies to clarify directionality and functionality (**Table 1**).

**Table 1:** Remodeling of the sympathetic nervous system (SNS) in the tumor microenvironment.

Cell types involved	Molecules involved	Cancer type	Biological effect	References
CAFs	NGF, β2-adrenergic receptor	Colorectal cancer	Bidirectional SNS-CAF crosstalk; activates ADRA2A/Gi-LATS-YAP pathway	(84)
Tumor cells	Exosome signaling	Head and neck cancer	Induce adrenergic reprogramming of sensory nerves	(103)
CINCs	BDNF	Adipose tissue	Support sympathetic process survival and growth	(94)
Lymphocytes	GM-CSF, NGF, cytokines	Prostate cancer	Stimulate sympathetic growth, regulate neurotransmitter gene expression	(95–98)
Macrophages (M2-like, SAMs)	SLIT3, ROBO1, PKA signaling	Adipose tissue, TME	Increase norepinephrine release, promote sympathetic growth	(100)
Endothelial cells	NGF receptors	Thermogenic adipose tissue	Promote neurovascular alignment and sympathetic sprouting	(93)

### 2.2 Remodeling of the parasympathetic nervous system in the TME

Parasympathetic nerves and specialized chemosensory cells (e.g., gastrointestinal tuft cells) release acetylcholine, signaling through nicotinic (nAChRs) and muscarinic (mAChRs) receptors on target cells to regulate biological functions and maintain homeostasis (104, 105). Immunohistochemical analysis of the parasympathetic marker vesicular acetylcholine transporter (VAChT) shows parasympathetic fiber innervation in various solid tumor TMEs, including prostate, breast, hepatocellular, gastric, and colorectal cancers (10, 27, 106–108).

Unlike sympathetic nerves, cholinergic signaling-mediated tumor phenotypes exhibit pronounced tissue specificity. Parasympathetic fibers are positive regulators in prostate and hepatocellular cancers. Compared to peritumoral normal tissue, these cancers show high expression of M1 muscarinic receptors, a G protein-coupled receptor subtype that couples to Gq/11 to activate phospholipase C and mobilize intracellular calcium. The density of parasympathetic fibers strongly correlates with poor clinical outcomes in these cancers (10, 108, 109). Parasympathetic nerves also exhibit pro-tumor effects in gastric (3, 27) and colorectal cancers (109). Genetic deletion or pharmacological inhibition of M3 muscarinic receptor (M3R) in gastric epithelial cells slows tumor growth and progression (3, 19). Similarly, low parasympathetic fiber density and reduced M3R or α9 nAChR expression in colorectal tumors correlate with favorable prognosis (110), while M3R stimulation increases tumor progression (111, 112). Mechanistically, cholinergic signaling in the TME promotes cancer cell proliferation, migration, invasion, and angiogenesis (113). Muscarinic signaling via M1R, M3R, and M5R enhances cancer cell viability and motility (114), facilitating metastasis. Beyond muscarinic receptors, nAChRsα7 and α9 are expressed in lung, colon, breast, and bladder cancers (115, 116). Their activation induces calcium-triggered autocrine release of growth factors, promoting proliferation (117). α7 nAChR-mediated cholinergic signaling also stimulates Ras-ERK-MAPK and JAK2 pathways, driving cancer cell proliferation and migration (117, 118).

Conversely, in breast and pancreatic cancers, parasympathetic fibers are tumor-suppressive, as low fiber density correlates with poor outcomes (80, 106, 119). Vagus nerve, the major parasympathetic component, links the brain and peripheral immune system via afferent and efferent fibers. Vagus-derived acetylcholine modulates immune cell cytokine release to regulate inflammatory phenotypes (120, 121). Accordingly, vagotomy, the surgical resection of parasympathetic and sensory nerves, accelerates cancer progression in breast and pancreatic cancers, where parasympathetic signaling exerts tumor-suppressive effects (80, 122, 123).

The tissue-specific phenotypes of parasympathetic nerves suggest their remodeling may be highly complex and dynamic. Cholinergic signaling-tumor cell crosstalk remains poorly understood in many solid tumor models, with current research focusing on acetylcholine interactions with neurotrophins and immune cells.

Acetylcholine binding to epithelial acetylcholine receptors can upregulate neurotrophin expression, strengthening parasympathetic-tumor interactions. For example, in gastric cancer models, M3R binding stimulates gastric epithelium, inducing NGF expression, which then targets TrkA receptors to trigger cholinergic neurite outgrowth (3, 19). This NGF feedback loop potentially stimulates colorectal carcinogenesis (19, 42).

Most immune cells (T lymphocytes, macrophages, dendritic cells, and natural killer cells) express α7nAChR. Thus, parasympathetic nerves, particularly the vagus, interact with tumor immunity, and immune cells are closely studied as potential drivers of parasympathetic differentiation and functional remodeling. For instance, vagal electrical stimulation or nicotine agonism of α7nAChR inhibits NF-κB transcriptional activity in human macrophages (124, 125), suppressing TNFα secretion (126). Macrophage-derived TNFα also plays a key role in pancreatic cancer (127). Vagal signaling also alters T lymphocyte function. In mouse models, unilateral cervical vagotomy transiently increases the release of immature CD4+CD8+ double-positive T cells from the thymus into peripheral circulation (128). In endotoxemic rat models, electrical stimulation of the left distal vagus nerve enhances splenic cytotoxic T lymphocyte activity (129).

Myeloid-derived suppressor cells (MDSCs), which suppress antitumor immunity by promoting regulatory T-cell proliferation (130), express nAChRs and are modulated by cholinergic signaling (131). Thus, parasympathetic (especially vagal) neuromodulation is a major factor influencing MDSC behavior in the TME. Consistent with parasympathetic tissue specificity, cholinergic-MDSC interactions exhibit opposing pro- or anti-tumor effects across models.

In breast cancer models, vagal electroacupuncture increases MDSCs in blood, spleen, and tumors (132), with acetylcholine exceeding levels achieved by vagal stimulation alone (133), suggesting other TME components participate in cholinergic-MDSC interactions. Further studies confirm immune cells form a positive feedback loop with the vagus via acetylcholine release (134, 135), regulating MDSC subtypes and numbers. Conversely, vagal stimulation increases splenic T-cell trefoil factor 2 (TFF2) expression, suppressing MDSC-producing myeloid progenitor proliferation and colon tumor growth (136) (**Table 2**).

**Table 2:** Remodeling of the parasympathetic nervous system (PNS) in the tumor microenvironment.

Cell types involved	Molecules involved	Cancer type	Biological effect	References
Parasympathetic nerve fibers	Acetylcholine, M1R/M3R, α7/α9 nAChR	Prostate, liver, gastric, colorectal cancer	Promote cancer cell proliferation, migration, invasion, angiogenesis	(3, 10, 19, 27, 107, 109, 110)
Parasympathetic nerve fibers	Acetylcholine, M3R	Breast, pancreatic cancer	Suppress tumor growth	(80, 106, 122, 123)
Gastric epithelial cells	NGF, TrkA receptor	Gastric cancer	Form cholinergic positive feedback loop, promote neurite outgrowth	(3, 19)
Immune cells	α7 nAChR	Multiple cancers	Inhibit NF-κB, regulate T cell and MDSC function	(124–129)
MDSCs	nAChR, cholinergic signaling	Breast, colon cancer	Bidirectional regulation of MDSCs; opposing effects across different models	(131–136)
Tumor cells	Acetylcholine, M3R	Colorectal, gastric cancer	Promote cell proliferation, EGFR activation, anti-apoptotic signaling	(26–28, 111)

### 2.3 Visceral neural plasticity in the TME

The understanding of visceral neural plasticity adds a brand-new dimension to sensory nerve remodeling in the TME. Visceral neural plasticity, linked with chronic visceral pain and hypersensitivity (137, 138), is well-documented in gastrointestinal inflammation and central nervous systems (139, 140). Locally, tumor and neuron-derived paracrine signals bidirectionally modulate peripheral sensory nerves, leading to hypersensitivity, sprouting, and perineural infiltration, leading to cancer pain (141–143).

Pancreatic tumors exhibit unparalleled neural plasticity, including increased density, hypertrophy, pancreatitis, and tumor infiltration of pancreatic plexuses (62, 85, 144, 145). The extent of neural invasion correlates with pancreatic neuropathy and pain severity (144). Pancreatic cancer patients show reduced sympathetic and cholinergic innervation (85), particularly in severe pancreatitis and abdominal pain. *In vitro*, pancreatic cancer tissue extracts induce neural plasticity, increasing density and hypertrophy (146). Thus, the TME is key to pancreatic neuropathy and plasticity (146). Mechanistic studies simulating pancreatic TME neurotrophic features identify NGF and artemin as critical neural remodeling factors (147). Artemin is a GDNF family neurotrophic factor. It signals through the GFRα3-RET receptor complex to promote neuronal survival and axon outgrowth. In pancreatic cancer, artemin enhances perineural invasion and stimulates the proliferation of peritumoral nerve fibers.

Breakthroughs in visceral neural plasticity mechanisms have emerged from gastric cancer models. In orthotopic gastric cancer mouse models, tumor cells overexpress NGF to recruit sensory nerves into the tumor. These nerves form synapse-like connections with cancer cells and establish bidirectional electrical communication. The sensory nerves then release calcitonin gene-related peptide (CGRP), which binds to its receptor on gastric cancer cells and drives cancer cell proliferation and metastasis (148).

This novel sensory remodeling links neuropathic pain and visceral neuropathy to altered innervation (85), suggesting neural remodeling modulation as a potential therapeutic strategy for tumor-related inflammation and pain. Supporting this, α7 nAChR activation alleviates pancreatitis-associated pain and inflammation in animal models (149). Besides, given the neural injury features in pancreatic cancer, activated glial cells possibly participate in visceral sensory remodeling and reinnervation (8, 85, 144). However, similar studies focusing on the cancer-related visceral plasticity remain scarce.

## 3 Expression remodeling of neural-related functional molecules in the TME: the neurotrophin family

The neurotrophin family includes NGF, BDNF, NT-3, and neurotrophin-4/5 (NT-4/5) (150). In 1991, TrkA was identified as NGF’s signaling receptor (151, 152), TrkB as BDNF and NT-4’s receptor (153–155), and TrkC as NT-3’s receptor. Trk receptor dimerization induces intracellular receptor trans-autophosphorylation, activating downstream pathways like Ras, Rac, PI3K, and PLC-γ1 (156).

Beyond Trk receptors, p75NTR, a tumor necrosis factor receptor family member, is a low-affinity NGF receptor (157) that binds all neurotrophins with similar affinity (158). p75NTR can activate Jun kinase and NF-κB pathways (159), participating in neuronal survival decisions and axon growth.

Although neurotrophins are primarily known for their neural effects, as multifunctional growth factors, they also play key roles in tumor-neural crosstalk. By directly affecting cancer cell proliferation, migration, and invasion, or indirectly influencing neural infiltration and differentiation, neurotrophins mediate tumor progression in various solid cancers, amplifying tumor-neural crosstalk. Moreover, detecting and quantifying neurotrophin levels aids tumor diagnosis and prognosis (20, 21), underscoring their significance in the TME.

The following sections detail the role of NGF and its receptors in specific cancer types.

### 3.1 Expression remodeling of NGF in the TME

NGF, a key neurotrophin, supports neuron survival (150, 160), regulates axon growth and synaptogenesis (161, 162), and influences neurotransmitter and neuropeptide synthesis (163–165). Discovered in the 1950s, NGF was among the first neurotrophins extensively studied for neural development and function (166). NGF is produced by various cells, including immune, endothelial, and neural tissues.

NGF is synthesized as proNGF (167, 168), which is processed into mature NGF. proNGF can bind p75NTR or sortilin (a vacuolar protein sorting 10 protein family member), exerting effects opposite to neurotrophins in neuronal development, injury-induced death, and synaptic plasticity (169, 170).

NGF receptors TrkA and p75NTR are closely associated with cancer cell proliferation, survival, and death (171). TrkA, NGF’s high-affinity receptor, triggers intracellular signaling (e.g., PI3K/Akt and MAPK/ERK pathways), promoting survival, proliferation, and differentiation. Conversely, NGF or proNGF binding to p75NTR induces apoptosis. p75NTR suppresses melanoma, prostate, bladder, and gastric cancer cell growth and hyperproliferation (172–174). Mechanistically, TrkA and p75NTR can compete or cooperate for NGF binding, depending on cellular context (175, 176). This receptor interplay fine-tunes NGF signaling, precisely controlling neuronal development, plasticity, and survival.

NGF pathway activation in cancer cells promotes aggressive growth, metastasis, and therapy resistance, becoming a key factor in various tumors. NGF’s dual role as an oncogenic factor and pro-apoptotic mediator potentially stems from its receptor complexity. Thus, analyzing NGF’s tumor-specific mechanisms and interactions with other TME components is crucial to understanding its plasticity.

#### 3.1.1 Breast cancer

NGF and its receptors are widely overexpressed in breast cancer, serving as diagnostic and prognostic markers (177–179) and potential therapeutic targets (180). NGF and proNGF activate breast cancer cell proliferation, migration, invasion, and angiogenesis via TrkA and NGFR/p75NTR pathways. NGF also interacts with cancer stem cells (CSCs), highlighting its functional plasticity in breast cancer.

NGF promotes breast cancer cell survival and proliferation (181, 182), and TrkA activation enhances triple-negative breast cancer cell proliferation, migration, and invasion (183). NGF’s pro-tumor effects rely on TrkA/β1-integrin/FAK/Src complex assembly, activating downstream effectors. By contrast, proNGF stimulates breast cancer invasion by binding sortilin and TrkA (184). Sequential formation of sortilin/TrkA/EphA2 complexes upon proNGF-receptor binding is observed, and dual targeting of TrkA and EphA2 significantly reduces primary tumor growth and brain metastasis in breast cancer models (185).

In breast cancer models, tumor-derived or exogenous NGF stimulates angiogenesis. NGF increases vascular endothelial growth factor (VEGF) secretion in endothelial and breast cancer cells (186), and TrkA binding upregulates matrix metalloproteinase 2 (MMP2) and nitric oxide synthase via PI3K and ERK pathways (186). NGF also induces matrix metalloproteinase 9 (MMP9) release, further promoting endothelial migration and angiogenesis (183), suggesting NGF and its pathways as potential anti-angiogenic targets (186).

CSCs, with self-renewal and therapy resistance, drive tumor initiation and metastasis (187). NGF and proNGF enrich CSCs in breast cancer cell lines (179), and NGF pretreatment promotes EMT in models (179), confirming NGF’s role in CSC regulation.

#### 3.1.2 Prostate cancer

In prostate cancer, TME interactions upregulate NGF and TrkA signaling, promoting metastasis, invasion, and neural invasion. CAFs activate YAP1/TEAD1 signaling, increasing tumor cell NGF secretion and neural invasion (22). Neural invasion-induced EMT suggests a feedback loop between neurons and prostate cancer cells (22). Moreover, in bone metastasis, E3 ubiquitin ligase FBXO22 upregulates macrophage NGF transcription, activating NGF/TrkA pathways to promote metastasis (188). Similarly, metastatic prostate tissues overexpress TRAF4 (189), correlating with NGF-induced gene expression (190). TrkA, a TRAF4 ubiquitination substrate, promotes prostate cancer cell invasion (190). Meanwhile, TrkA mediates castration-resistant prostate cancer (CRPC) cell proliferation, invasion, and EMT (191), highlighting TrkA as a novel CRPC biomarker (191).

Androgens also influence hormone-sensitive tumor cells via NGF receptor crosstalk (192). Dehydroepiandrosterone (DHEA) modulates cell fate and activates NGF receptors (TrkA and p75NTR) in neurons and tumor cells (193), offering new insights into prostate cancer pathophysiology.

Notably, NGF promotes perivascular nerve reinnervation in prostate models, regulating vascular tone and blood flow to suppress tumor growth (194). These conflicting findings necessitate further mechanistic studies.

Prostate cancer cells secrete proNGF to induce axonogenesis (20), suggesting proNGF as a neural invasion driver and high-risk prostate cancer biomarker (20). In contrast, p75NTR suppresses prostate cancer progression (195), with expression inversely correlating with malignancy (196). p75NTR activation inhibits NGF-induced prostate cancer cell migration (195).

#### 3.1.3 Digestive system tumors

In gastric, colon, and pancreatic cancers, tumor tissues overexpress NGF, mediating neural invasion and correlating with proliferation and metastasis. NGF reduces serum deprivation-induced apoptosis in colon cancer cells (192). Recent studies have definitively demonstrated that nerve fibers within colon tumors are recruited into the tumor interior through NGF secreted by cancer cells (148). Besides, pancreatic cancer tissues exhibit higher NGF levels than adjacent tissues (197), explaining their high invasiveness and neural infiltration rates (81, 198). In 3D migration assays, pancreatic cancer cells show greater neurotropic migration, and their conditioned media increase neurite density (198). Inhibiting NGF or its receptors reduces pancreatic cancer cell proliferation and migration *in vitro* (199). Further research proves that HGF/c-Met signaling activates mTOR/NGF axis to promote pancreatic PNI (200). In gastric cancer, cholinergic stimulation induces NGF expression, expanding enteric nerves and promoting progression (19).

Controversially, while some studies link NGF overexpression to poor gastric cancer prognosis, others find higher NGF expression in normal gastric tissue (201). p75NTR downregulates MMP9 via NF-κB, inhibiting gastric cancer invasion and metastasis (174), suggesting NGF and receptors may uniquely regulate apoptosis via Ras or Raf pathways (201).

NGF mediates tumor-TME interactions in digestive cancers. In gastric cancer, SNRPA is overexpressed and correlates with tumor size and progression (202). SNRPA is a core component of the U1 small nuclear ribonucleoprotein complex. This complex recognizes the 5’ splice site and catalyzes the early steps of pre-messenger RNA splicing. SNRPA is essential for NGF-mediated gastric cancer cell growth (202, 203). In colon cancer, Schwann cell-derived NGF promotes colon cancer cell proliferation and Schwann cell migration (74). Colon cancer exosomal miR-21-5p increases Schwann cell NGF transcription (74), activating TrkA/ERK/ELK1/ZEB1 signaling in cancer cells to further upregulate miR-21-5p, forming an NGF-mediated feedback loop (74).

### 3.2 Remodeling of BDNF in the TME

BDNF, the most studied astrocyte-derived neurotrophin, and its receptor TrkB promote tumor progression in central nervous system (CNS) cancers by supporting cell survival (204, 205), inhibiting apoptosis (205–207), and enhancing migration and invasion (208–211). BDNF/TrkB also strongly induces angiogenesis (212, 213) by increasing HIF-1α, upregulating VEGF (212) and TrkB (214), and recruiting endothelial progenitor cells (215). Besides, BDNF/TrkB overexpression correlates with therapy resistance (216).

As the precursor of BDNF (217), proBDNF inhibits glioma apoptosis via Trk receptors (218), while BDNF exhibits tumor-suppressive effects (219), suggesting balanced BDNF phenotypes regulate glioma growth.

In peripheral solid tumors, BDNF/TrkB demonstrates diverse pro-tumor effects (220, 221), though mechanisms remain less clear than in CNS tumors, warranting further research.

#### 3.2.1 Gastric cancer

BDNF/TrkB upregulation is a gastric cancer driver and therapeutic target (222). TrkB overexpression in gastric cancer correlates with poor outcomes (223). TrkB inhibition reduces tumor burden and peritoneal metastasis in models (222). BDNF/TrkB enhances gastric cancer-osteoblast niche interactions, promoting bone metastasis (224).

#### 3.2.2 Colon cancer

Colon tumors overexpress BDNF/TrkB at transcriptional and protein levels (225), with co-expression linked to liver and peritoneal metastasis (226, 227). TrkB induces EMT via Twist/Snail transcription factors (228), promoting invasion and metastasis. TrkB inversely correlates with E-cadherin and positively with vimentin (229), suggesting anti-apoptotic roles.

BDNF/TrkB contributes to colon cancer therapy resistance. Cetuximab (anti-EGFR) often faces resistance (230, 231), and BDNF/TrkB-EGFR crosstalk reveals resistance mechanisms (232).

#### 3.2.3 Pancreatic cancer

Compared to NGF, the role of BDNF in pancreatic cancer is less extensively characterized. Current evidence shows that TrkB overexpression marks poor prognosis and invasiveness in PDAC (233), correlating with neural invasion and liver metastasis latency (233). Further studies are needed to fully elucidate the BDNF-TrkB signaling axis in pancreatic cancer.

#### 3.2.4 Breast cancer

BDNF is aberrantly expressed in breast cancer (234), promoting survival and growth (234). BDNF/TrkB induces EMT, enhancing migration and invasion (229). EMT, a process where epithelial cells lose junctions and undergo cytoskeletal remodeling (235), is stimulated by BDNF/TrkB in breast cancer (236, 237). BDNF/TrkB activation reduces E-cadherin (238), promoting invasion and metastasis (239). TrkB downregulates tumor suppressors Runx3 and Keap1, directly inducing metastasis (240).

In breast TME, BDNF may induce CSC self-renewal. Recurrent triple-negative breast cancer exhibits BDNF/TrkB-expressing CSCs and paracrine signaling (237).

### 3.3 Remodeling of other neurotrophins in the TME

Compared to NGF and BDNF, NT-3 and NT-4/5’s roles in peripheral tumors are less understood. In melanoma, TrkC overexpression or NT-3 treatment promotes proliferation, migration, and invasion (241, 242), particularly in brain metastasis, where cancer cell invasion parallels NT secretion (242–244).

Conversely, TrkC is a conditional tumor suppressor in colon cancer (245), with downregulated expression (246)and promoter methylation silencing TrkC. NT-3 loss precedes TrkC loss, conferring survival advantages (246).

The completely opposite functional remodeling of NT-3 in different TME highlight neurotrophin complexity, necessitating further research.

## 4 Tumor treatment strategies based on neural remodeling

Over the past two decades, extensive preclinical and clinical evidence has elucidated the role of the nervous system in promoting cancer growth (247–249). Concurrently, given the structural and functional remodeling characteristics of the nervous system in the TME, the exploration of neuroscience-based drugs for antitumor therapy has garnered increasing attention in recent years (250). Considering the features of tumor-neural crosstalk, we summarize tumor treatment strategies based on neural remodeling from multiple perspectives, including blocking axonogenesis, remodeling autonomic nerve differentiation phenotypes, and inhibiting neurotrophic factor signaling pathways. Finally, we also highlight the significance of combination therapy strategies in antitumor treatment, emphasizing potential therapeutic opportunities and challenges inspired by preclinical and preliminary clinical data.

### 4.1 Tumor treatment strategies targeting axonogenesis

In various solid tumor models, increased nerve density has been shown to correlate strongly with tumor initiation and progression. Additionally, quantifying neural infiltration through pathological examination of clinical specimens can help determine tumor grade and predict patient prognosis and treatment response (10, 251). Therefore, reducing nerve density by targeting axonogenesis in the TME is regarded as an ideal tumor treatment strategy. Approaches include surgical denervation at the macroscopic level and pharmacological inhibition of axonogenesis at the microscopic level. Exogenous drug options include neurotoxic agents and antibodies or inhibitors targeting neurotrophic factor families.

The clinical value of vagotomy has been widely discussed in denervation surgery. Studies have confirmed that unilateral or bilateral vagotomy in animal models can inhibit gastric cancer metastasis by reducing Wnt signaling (3). Conversely, consistent with the tissue-specific effects of parasympathetic nerves in different TMEs, vagotomy promotes cancer progression in pancreatic cancer (80). Similarly, the incidence of distant adrenal metastasis in breast cancer increases after vagotomy (123). Further research suggests that the antitumor effect of the vagus nerve may result from upregulated TNF-α expression in the TME (252), a cholinergic anti-inflammatory pathway closely associated with α7 nAChR (253).

However, denervation surgery is notably nonspecific, as surgical resection severs all nerve fibers in the surrounding nerves, including those critical for maintaining normal organ function. Thus, surgical intervention to modulate nerve density may cause unnecessary side effects and adversely impact comprehensive tumor treatment.

Compared to nonsurgical approaches, neurotoxic drugs can target specific neuronal subtypes, demonstrating advantages in clinical denervation therapy. A Phase I clinical trial tested Botulinum toxin-mediated denervation in 26 patients with prostate cancer (NCT00067496). Botox injection reduced nerve density in the prostate TME and induced tumor cell apoptosis (3). Additionally, Botox-mediated denervation has been tested in a Phase I clinical trial for prostate cancer (254). Results showed that Botox injection reduced nerve density in the prostate TME and induced tumor cell apoptosis (254), suggesting its applicability for denervation in solid tumors. However, Botox and other neurotoxic agents, such as 6-hydroxydopamine (6-OHDA), can cross the blood-brain barrier and cause central nervous system toxicity. Thus, these drugs may also affect non-neuronal cells and tissues, exhibiting specificity limitations.

Compared to surgical or chemical denervation, targeting neurotrophic factors closely associated with tumor axonogenesis to reduce nerve infiltration may be a more suitable strategy (19, 81, 255). This approach preserves normal organ innervation and avoids non-neuronal toxicity, offering clear advantages for clinical translation. Among neurotrophic factors, nerve growth factor (NGF) has the most pronounced effect on inducing nascent axons. Accordingly, denervation strategies targeting NGF and its specific receptors show great potential in tumor therapy. Anti-NGF blocking antibodies or nanoparticle-delivered NGF siRNA have been shown to reduce tumor growth and metastasis in animal models (256). The success of nanoparticle-based strategies highlights the importance of overcoming stromal barriers and minimizing off-target toxicity. Advanced delivery systems, such as tumor microenvironment-responsive nanoparticles that penetrate desmoplastic stroma via charge reversal or transcytosis mechanisms, offer promising solutions to achieve precise intratumoral neuromodulation while avoiding systemic side effects (257, 258). Additionally, drugs such as entrectinib, larotrectinib, and cabozantinib, which target the BDNF/TrkB pathway (259–261), provide new insights into neurotrophic factor-based denervation therapy by blocking axon regeneration mediated by this pathway.

### 4.2 Tumor treatment strategies targeting autonomic nervous system remodeling

In multiple tumor models, the TME has been shown to induce functional remodeling of the autonomic nervous system. Specifically, sympathetic β-adrenergic signaling accelerates cancer progression, whereas parasympathetic cholinergic signaling exhibits tissue-specific effects. Pharmacological strategies targeting autonomic remodeling primarily focus on modulating adrenergic and cholinergic signaling. Additionally, genetic tools such as optogenetics offer novel approaches for nerve-mediated tumor therapy (262, 263).

#### 4.2.1 Pharmacological strategies targeting adrenergic signaling

Among adrenergic signaling-targeted therapies, β-adrenergic receptor (β-AR) antagonists are the most extensively studied. Research has also revealed the role of α-adrenergic receptor (α-AR) antagonists in modulating sympathetic signaling to influence tumor progression (264).

Propranolol is the most thoroughly investigated β-AR antagonist for tumor therapy. In breast cancer models, *in vitro* studies demonstrate that propranolol inhibits tumor cell proliferation, invasion, migration, and angiogenesis (87, 265). A randomized trial confirmed that preoperative oral propranolol reduces levels of metastasis-associated biomarkers in breast cancer (266). Further studies link propranolol’s antimetastatic effects to miR-499-5p and Sox6 (267). Propranolol’s antitumor efficacy has also been validated in other solid tumor models. In colon cancer, it activates CD8+ T cells and suppresses AKT/MAPK signaling to inhibit tumor progression (268).

While the antitumor effects of β-AR antagonists like propranolol are well-established, their clinical efficacy requires direct evaluation in randomized trials. Multiple clinical studies highlight the significant role of β-AR antagonists in reducing breast cancer metastasis, recurrence, and mortality (269–271). Retrospective analyses further associate β-AR antagonist use with improved recurrence-free survival in breast cancer patients (272). The therapeutic potential of β-AR antagonists has also been assessed in other peripheral tumors. Cohort studies indicate that β-AR antagonist therapy correlates with reduced risk of malignant melanoma progression (273), and treated patients exhibit lower recurrence rates and prolonged survival (274, 275). Besides, β-AR antagonist therapy can enhance the survival rate of PDAC patients (276).

Similar to β-AR antagonists, α-AR antagonists show promise in tumor therapy. Naftopidil, for instance, exerts antiproliferative effects via G0/G1 cell cycle arrest (277) and reduces tumor volume in multiple solid tumor models (278, 279). It also exhibits antiangiogenic effects, significantly decreasing microvessel density in renal and prostate cancer models (280). Clinical evidence supports naftopidil’s antitumor properties. A retrospective study notes reduced prostate cancer incidence in patients receiving naftopidil (281), and animal studies report good tolerance (282). Other α-AR antagonists, such as phenoxybenzamine, inhibit TrkB/Akt signaling to suppress malignant glioma proliferation (283).

Given their widespread use in cardiovascular diseases, adrenergic receptor antagonists offer advantages in cancer therapy, including lower development costs, reduced clinical trial risks, and shorter timelines (284). However, clinical translation faces challenges, such as side effects (e.g., fatigue, dyspnea, dizziness, metabolic disturbances) due to the ubiquitous expression of adrenergic receptors. Contraindications like asthma, bradycardia, and hypertension may further limit their applicability in cancer patients with complex health conditions.

#### 4.2.2 Pharmacological strategies targeting cholinergic signaling

In the TME, parasympathetic-derived cholinergic signaling promotes tumor invasion and metastasis in multiple peripheral tumors, including lung (285), gastric (286), and prostate cancers (10). Cholinergic antagonists like atropine reverse these protumor phenotypes (287). For example, in prostate adenocarcinoma, elevated CHRM1 expression is countered by pharmacological or genetic blockade such as CHRM1 gene silencing with siRNA, reducing cholinergic-mediated invasion and metastasis (10). Galantamine, an acetylcholine esterase inhibitor, reduces aberrant crypt foci, which are the earliest precursor lesions of colorectal cancer, and restores colon architecture in animal models (288). In intestinal tumors, the anticholinergic scopolamine butylbromide is less effective than genetic M3 receptor deletion, likely due to incomplete receptor blockade (111).

Notably, parasympathetic remodeling exhibits tissue-specific effects, necessitating model-specific therapeutic approaches. For instance, vagus nerve activation via semapimod reduces primary tumor growth and metastasis in breast cancer models (289, 290).

Anticholinergic anesthetics have also been explored for cancer prevention, inhibiting mammary tumorigenesis (291) and reducing recurrence-associated markers like NETosis and matrix metalloproteinase 3 (MMP3) (292).

#### 4.2.3 Optogenetics-mediated autonomic nerve remodeling in tumor therapy

Optogenetic tools, which reversibly modulate cellular functions via light-sensitive proteins, offer novel therapeutic strategies (293). For example, to address safety concerns in CAR-T cell therapy (294, 295), optogenetically engineered T cells enable precise, light-controlled activation to target tumors while minimizing off-target effects (296). In animal models, optogenetic stimulation of colonic sympathetic fibers reduces CD45+ cell infiltration, modulating immune responses and alleviating colitis (297). Such localized control of sympathetic signaling and immune modulation may inspire new cancer therapies.

### 4.3 Neurotrophic factor-based tumor treatment strategies

Inhibiting neurotrophic factor signaling is another emerging area of interest. High-throughput screening is ideal for identifying ligands and receptors (298, 299). Most strategies target the NGF/TrkA pathway, using blocking antibodies, small-molecule inhibitors, or peptides (300). Systemic anti-NGF antibody administration shows no significant neuronal or cognitive effects (301), underscoring its therapeutic potential (301). TrkA inhibitors like PHA-848125 (a dual CDK/TrkA inhibitor) exhibit preclinical efficacy in glioma, supporting Phase II trials (302, 303).

Beyond antitumor effects, NGF/TrkA-targeted therapies alleviate cancer pain (304, 305). A randomized placebo-controlled trial of 155 patients with bone metastasis pain (NCT00545129) showed that the anti-NGF antibody tanezumab significantly reduced pain scores (306). The humanized anti-NGF antibody tanezumab, tested for chronic pain (301), reduces bone metastasis-induced pain (306). Similarly, anti-NGF antibodies mitigate pain and bone destruction in prostate cancer models (307, 308), and the TrkA-specific antibody MNAC13 shows analgesic effects (309). Thus, neurotrophic factor-targeted therapies uniquely address both cancer progression and pain.

However, the dual synergistic/competitive binding of NGF to TrkA and p75NTR (175, 176) necessitates model-specific strategies. While TrkA inhibitors improve survival in TrkA fusion-positive malignancies (261, 310), their efficacy in other solid tumors remains unproven. Off-target effects due to broad tyrosine kinase affinity also pose challenges (311).

Targeting other neurotrophic factors, such as BDNF/TrkB, shows promise. In gastric cancer models, BDNF/TrkB blockade reduces innervation and tumor growth (222). The pan-tyrosine kinase inhibitor ry-470 also alleviates cancer pain and neural remodeling in sarcoma models (312).

### 4.4 Combination therapies

Combination therapies are increasingly used to enhance efficacy and reduce toxicity. Targeting tumor-neural interactions is critical for overcoming resistance to chemotherapy and immunotherapy.

#### 4.4.1 Immunotherapy combinations

Immunotherapy demonstrates broad therapeutic potential in oncology, yet suboptimal response rates in multiple solid tumors limit patient benefit. The development of combination therapies targeting neural remodeling signatures within the tumor microenvironment represents a novel strategy to overcome immune resistance. Studies reveal significantly reduced anti-PD-1 response rates in skin, gastric, and melanoma cancer patients exhibiting neuropathic alterations—a phenomenon attributed to chronic inflammatory microenvironments induced by injured nerves (313). Since adrenergic signaling modulates immune responses and inflammation, adrenergic antagonists may enhance efficacy. Clinical studies evaluate combinations like propranolol with anti-PD-1 (pembrolizumab) in melanoma, showing improved outcomes (314, 315). Propranolol plus anti-CTLA4 also benefits sarcoma patients (316). Retrospective analyses suggest adrenergic antagonists enhance anti-PD-1 efficacy in melanoma (317). Downregulating adrenergic signaling in CAR-T cells enhances cytotoxicity against prostate tumors (318). However, the complexity of adrenergic-immune crosstalk warrants further validation.

#### 4.4.2 Chemotherapy combinations

Adrenergic signaling affects chemosensitivity. For example, norepinephrine upregulates drug efflux genes (e.g., ABCG2) in cholangiocarcinoma (319) and induces cisplatin resistance via ADRB2/Akt/ABCG2 in head and neck squamous cell carcinoma (320). Similarly, adrenergic signaling contributes to anthracycline resistance (321). Thus, adrenergic blockade may sensitize tumors to chemotherapy.

Clinical studies support propranolol-chemotherapy combinations. In triple-negative breast cancer, propranolol reduces progression and mortality with anthracyclines (321). In colon cancer, it disrupts hypoxia adaptation by inhibiting HIF1α/CAIX, enhancing irinotecan efficacy and immune responses (322, 323). Other adrenergic antagonists, like ICI 118551 (β2-AR antagonist) with gemcitabine (324) or nebivolol with erdafitinib (325), show synergistic effects. Naftopidil enhances docetaxel cytotoxicity in prostate cancer (279) and combines with trametinib to induce apoptosis in ovarian cancer organoids (326).

## 5 Conclusions

In 2011, a seminal review outlined ten hallmarks of cancer (327, 328), including sustained proliferative signaling, evasion of growth suppressors, resistance to cell death, replicative immortality, induction of angiogenesis, activation of invasion and metastasis, tumor-promoting inflammation, genome instability and mutation, reprogrammed cellular metabolism, and immune evasion. With the emergence of cancer neuroscience as a distinct field, “neuron signaling” has been proposed as an additional hallmark, reflecting the impact of tumor-nerve crosstalk on cancer progression (329).

This review has systematically elucidated three core aspects of neural remodeling within the tumor microenvironment. The first is structural remodeling. Tumors secrete neurotrophins such as nerve growth factor and brain-derived neurotrophic factor to stimulate axonogenesis, while simultaneously inducing Schwann cell reprogramming toward a repair-like phenotype, forming tumor-activated Schwann cell tracks that guide tumor cell migration (66). The second is autonomic nerve remodeling. The sympathetic nervous system promotes tumor progression by releasing norepinephrine and activating β-adrenergic signaling (84), whereas the parasympathetic nervous system exerts tissue-specific effects: it promotes tumor growth in gastric and colorectal cancers (19) but suppresses tumor progression in breast and pancreatic cancers (80). The third is neurotrophin functional remodeling. Nerve growth factor, brain-derived neurotrophic factor, NT-3, and NT-4/5 are dysregulated in the tumor microenvironment and activate downstream signaling pathways including PI3K/Akt and MAPK/ERK via Trk receptors and p75NTR, directly enhancing cancer cell proliferation, survival, and invasion (19, 81, 177).

Neural remodeling exhibits marked heterogeneity across different cancer types, offering multiple entry points for precision therapy. In breast cancer, β-adrenergic receptor antagonists such as propranolol have demonstrated clinical value in inhibiting metastasis and improving prognosis (266). In gastric and colorectal cancers, targeting muscarinic acetylcholine receptors or the nerve growth factor-TrkA signaling axis suppresses tumor progression, and vagotomy has been shown to be effective in animal models (3). In prostate cancer, Botulinum toxin-mediated denervation has entered clinical trials, and targeting nerve growth factor-TrkA signaling also shows promise (10). In pancreatic cancer, artemin and its receptor GFRα3 have emerged as novel therapeutic targets due to their critical roles in perineural invasion and neuropathic pain (147). In addition, drugs targeting neurotrophin signaling pathways, including anti-nerve growth factor antibodies and Trk inhibitors, not only suppress tumor growth but also alleviate cancer-related pain (306).

A key dimension of tumor-nerve crosstalk lies in the bidirectional interaction between peripheral nerves and immune cells within the tumor microenvironment. Sympathetic neurotransmitters such as norepinephrine modulate macrophage polarization, T cell function, and myeloid-derived suppressor cell activity, thereby shaping antitumor immunity (88). Conversely, immune cells including macrophages and lymphocytes secrete neurotrophins and cytokines that promote sympathetic and parasympathetic nerve remodeling (94). This neuro-immune crosstalk may be exploited to enhance the efficacy of cancer immunotherapy.

Despite these advances, significant technical limitations remain. Conventional histological approaches lack sufficient sensitivity to characterize the fine architecture of nerve fibers or to localize low-abundance signaling molecules within axons. Emerging tools including spatial transcriptomics, single-cell analysis, and lineage tracing will be essential to map tumor-nerve interactions across multiple scales. Overcoming the physical and pharmacological barriers to drug delivery, as well as deciphering how the acidic and hypoxic TME modulates neural excitability, represent key frontiers for future research. Addressing these challenges will clarify the mechanisms of tumor-induced neural remodeling and ultimately pave the way for precision cancer therapies that target the nervous system.

## Glossary

α-AR: α-adrenergic receptor
6-OHDA: 6-hydroxydopamine
ARTN: Artemin
BDNF: Brain-derived neurotrophic factor
Botox: Botulinum toxin
β-AR: β-adrenergic receptor
CAFs: Cancer-associated fibroblasts
CCL2: C-C Motif Chemokine Ligand 2
CGRP: Calcitonin gene-related peptide
CINCs: Cold-induced neuroimmune cells
CNS: Central nervous system
CRPC: Castration-resistant prostate cancer
CSCs: Cancer stem cells
CXCL2: C-X-C Motif Chemokine Ligand 2
DCX: Doublecortin
DHEA: Dehydroepiandrosterone
ECM: Extracellular matrix
EMT: Epithelial-mesenchymal transition
GDNF: Glial Cell Derived Neurotrophic Factor
GFAP: Glial Fibrillary Acidic Protein
GFRα3: GDNF Family Receptor Alpha 3
GM-CSF: Granulocyte-macrophage colony-stimulating factor
IL-1α: Interleukin 1 Alpha
IL-1β: Interleukin 1 beta
IL-6: Interleukin 6
IL-8: Interleukin 8
MDSCs: Myeloid-derived suppressor cells
MMP2: Metalloproteinase 2
MMP3: Metalloproteinase 3
MMP9: Metalloproteinase 9
NGF: Nerve growth factor
NT-3: Neurotrophin-3
NT-4/5: Neurotrophin-4/5
PDAC: Pancreatic ductal adenocarcinoma
p75NTR: p75 neurotrophin receptor
PKA: Protein kinase A
PNI: Perineural invasion
PNS: Peripheral nervous system
SAMs: Sympathetic-associated macrophages
SC-EVs: Schwann cell-derived extracellular vesicles
SLIT2: Slit Guidance Ligand 2
SNS: Sympathetic nervous system
TAST: Tumor-activated Schwann cell tracks
TFF2: T-cell trefoil factor 2
TME: Tumor microenvironment
TNF-α: Tumor necrosis factor alpha
VAChT: Vesicular acetylcholine transporter
VEGF: Vascular endothelial growth factor.

## References

[1] Kumar A Brockes JP . Nerve dependence in tissue, organ, and appendage regeneration. Trends Neurosci. (2012) 35:691–9. doi: 10.1016/j.tins.2012.08.003. PMID: 22989534

[2] Boilly B Faulkner S Jobling P Hondermarck H . Nerve dependence: From regeneration to cancer. Cancer Cell. (2017) 31:342–54. doi: 10.1016/j.ccell.2017.02.005. PMID: 28292437

[3] Zhao CM Hayakawa Y Kodama Y Muthupalani S Westphalen CB Andersen GT . Denervation suppresses gastric tumorigenesis. Sci Transl Med. (2014) 6:250ra115. doi: 10.1126/scitranslmed.3009569. PMID: 25143365 PMC4374618

[4] Stopczynski RE Normolle DP Hartman DJ Ying H DeBerry JJ Bielefeldt K . Neuroplastic changes occur early in the development of pancreatic ductal adenocarcinoma. Cancer Res. (2014) 74:1718–27. doi: 10.1158/0008-5472.can-13-2050. PMID: 24448244 PMC4036226

[5] Ayala GE Dai H Powell M Li R Ding Y Wheeler TM . Cancer-related axonogenesis and neurogenesis in prostate cancer. Clin Cancer Res. (2008) 14:7593–603. doi: 10.1158/1078-0432.ccr-08-1164. PMID: 19047084

[6] Yaniv D Mattson B Talbot S Gleber-Netto FO Amit M . Targeting the peripheral neural-tumour microenvironment for cancer therapy. Nat Rev Drug Discov. (2024) 23:780–96. doi: 10.1038/s41573-024-01017-z. PMID: 39242781 PMC12123372

[7] Fagan JJ Collins B Barnes L D'Amico F Myers EN Johnson JT . Perineural invasion in squamous cell carcinoma of the head and neck. Arch Otolaryngology--Head Neck Surg. (1998) 124:637–40. doi: 10.1001/archotol.124.6.637. PMID: 9639472

[8] Bockman DE Buchler M Beger HG . Interaction of pancreatic ductal carcinoma with nerves leads to nerve damage. Gastroenterology. (1994) 107:219–30. doi: 10.1016/0016-5085(94)90080-9. PMID: 8020665

[9] Nagakawa T Kayahara M Ohta T Ueno K Konishi I Miyazaki I . Patterns of neural and plexus invasion of human pancreatic cancer and experimental cancer. Int J Pancreatology. (1991) 10:113–9. doi: 10.1007/bf02924114. PMID: 1660909

[10] Magnon C Hall SJ Lin J Xue X Gerber L Freedland SJ . Autonomic nerve development contributes to prostate cancer progression. Science. (2013) 341:1236361. doi: 10.1126/science.1236361. PMID: 23846904

[11] Albo D Akay CL Marshall CL Wilks JA Verstovsek G Liu H . Neurogenesis in colorectal cancer is a marker of aggressive tumor behavior and poor outcomes. Cancer. (2011) 117:4834–45. doi: 10.1002/cncr.26117. PMID: 21480205

[12] Raju B Haug SR Ibrahim SO Heyeraas KJ . Sympathectomy decreases size and invasiveness of tongue cancer in rats. Neuroscience. (2007) 149:715–25. doi: 10.1016/j.neuroscience.2007.07.048. PMID: 17916410

[13] Huang D Su S Cui X Shen X Zeng Y Wu W . Nerve fibers in breast cancer tissues indicate aggressive tumor progression. Medicine. (2014) 93:e172. doi: 10.1097/md.0000000000000172. PMID: 25501061 PMC4602796

[14] Partecke LI Speerforck S Käding A Seubert F Kühn S Lorenz E . Chronic stress increases experimental pancreatic cancer growth, reduces survival and can be antagonised by beta-adrenergic receptor blockade. Pancreatology. (2016) 16:423–33. doi: 10.1016/j.pan.2016.03.005. PMID: 27083074

[15] Shao JX Wang B Yao YN Pan ZJ Shen Q Zhou JY . Autonomic nervous infiltration positively correlates with pathological risk grading and poor prognosis in patients with lung adenocarcinoma. Thorac Cancer. (2016) 7:588–98. doi: 10.1111/1759-7714.12374. PMID: 27766778 PMC5193011

[16] Zahalka AH Arnal-Estapé A Maryanovich M Nakahara F Cruz CD Finley LWS . Adrenergic nerves activate an angio-metabolic switch in prostate cancer. Sci (New York NY). (2017) 358:321–6. doi: 10.1126/science.aah5072. PMID: 29051371 PMC5783182

[17] Sinha S Fu YY Grimont A Ketcham M Lafaro K Saglimbeni JA . PanIN neuroendocrine cells promote tumorigenesis via neuronal cross-talk. Cancer Res. (2017) 77:1868–79. doi: 10.1158/0008-5472.can-16-0899. PMID: 28386018 PMC5471615

[18] Mauffrey P Tchitchek N Barroca V Bemelmans AP Firlej V Allory Y . Progenitors from the central nervous system drive neurogenesis in cancer. Nature. (2019) 569:672–8. doi: 10.1038/s41586-019-1219-y. PMID: 31092925

[19] Hayakawa Y Sakitani K Konishi M Asfaha S Niikura R Tomita H . Nerve growth factor promotes gastric tumorigenesis through aberrant cholinergic signaling. Cancer Cell. (2017) 31:21–34. doi: 10.1016/j.ccell.2016.11.005. PMID: 27989802 PMC5225031

[20] Pundavela J Demont Y Jobling P Lincz LF Roselli S Thorne RF . ProNGF correlates with Gleason score and is a potential driver of nerve infiltration in prostate cancer. Am J Pathol. (2014) 184:3156–62. doi: 10.1016/j.ajpath.2014.08.009. PMID: 25285721

[21] Pundavela J Roselli S Faulkner S Attia J Scott RJ Thorne RF . Nerve fibers infiltrate the tumor microenvironment and are associated with nerve growth factor production and lymph node invasion in breast cancer. Mol Oncol. (2015) 9:1626–35. doi: 10.1016/j.molonc.2015.05.001. PMID: 26009480 PMC5528785

[22] Shen T Li Y Wang D Su Y Li G Shang Z . YAP1-TEAD1 mediates the perineural invasion of prostate cancer cells induced by cancer-associated fibroblasts. Biochim Biophys Acta Mol Basis Dis. (2022) 1868:166540. doi: 10.1016/j.bbadis.2022.166540. PMID: 36100154

[23] Banh RS Biancur DE Yamamoto K Sohn ASW Walters B Kuljanin M . Neurons release serine to support mRNA translation in pancreatic cancer. Cell. (2020) 183:1202–1218.e25. doi: 10.1016/j.cell.2020.10.016. PMID: 33142117 PMC8100789

[24] Marlin MC Li G . Biogenesis and function of the NGF/TrkA signaling endosome. Int Rev Cell Mol Biol. (2015) 314:239–57. 10.1016/bs.ircmb.2014.10.002 PMC4307610 25619719

[25] Allen JK Armaiz-Pena GN Nagaraja AS Sadaoui NC Ortiz T Dood R . Sustained adrenergic signaling promotes intratumoral innervation through BDNF induction. Cancer Res. (2018) 78:3233–42. doi: 10.1158/0008-5472.can-16-1701. PMID: 29661830 PMC6004256

[26] Cheng K Samimi R Xie G Shant J Drachenberg C Wade M . Acetylcholine release by human colon cancer cells mediates autocrine stimulation of cell proliferation. Am J Physiol Gastrointest Liver Physiol. (2008) 295:G591–7. doi: 10.1152/ajpgi.00055.2008. PMID: 18653726 PMC2536781

[27] Wang L Zhi X Zhang Q Wei S Li Z Zhou J . Muscarinic receptor M3 mediates cell proliferation induced by acetylcholine and contributes to apoptosis in gastric cancer. Tumour Biol. (2016) 37:2105–17. doi: 10.1007/s13277-015-4011-0. PMID: 26346168

[28] Yu H Xia H Tang Q Xu H Wei G Chen Y . Acetylcholine acts through M3 muscarinic receptor to activate the EGFR signaling and promotes gastric cancer cell proliferation. Sci Rep. (2017) 7:40802. doi: 10.1038/srep40802. PMID: 28102288 PMC5244394

[29] Zhong LR Estes S Artinian L Rehder V . Acetylcholine elongates neuronal growth cone filopodia via activation of nicotinic acetylcholine receptors. Dev Neurobiol. (2013) 73:487–501. doi: 10.1002/dneu.22071. PMID: 23335470

[30] Jobling P Pundavela J Oliveira SM Roselli S Walker MM Hondermarck H . Nerve-cancer cell cross-talk: A novel promoter of tumor progression. Cancer Res. (2015) 75:1777–81. doi: 10.1158/0008-5472.can-14-3180. PMID: 25795709

[31] Zhang X Zhang Y He Z Yin K Li B Zhang L . Chronic stress promotes gastric cancer progression and metastasis: an essential role for ADRB2. Cell Death Dis. (2019) 10:788. doi: 10.1038/s41419-019-2030-2. PMID: 31624248 PMC6797812

[32] Perrone MG Notarnicola M Caruso MG Tutino V Scilimati A . Upregulation of beta3-adrenergic receptor mRNA in human colon cancer: a preliminary study. Oncology. (2008) 75:224–9. doi: 10.1159/000163851. PMID: 18852493

[33] Akil H Perraud A Mélin C Jauberteau MO Mathonnet M . Fine-tuning roles of endogenous brain-derived neurotrophic factor, TrkB and sortilin in colorectal cancer cell survival. PloS One. (2011) 6:e25097. doi: 10.1371/journal.pone.0025097. PMID: 21966426 PMC3180371

[34] Hisasue S Kato R Sato Y Suetomi T Tabata Y Tsukamoto T . Cavernous nerve reconstruction with a biodegradable conduit graft and collagen sponge in the rat. J Urol. (2005) 173:286–91. doi: 10.1097/01.ju.0000141578.84536.80. PMID: 15592098

[35] Twardowski T Fertala A Orgel JP San Antonio JD . Type I collagen and collagen mimetics as angiogenesis promoting superpolymers. Curr Pharm Des. (2007) 13:3608–21. doi: 10.2174/138161207782794176. PMID: 18220798

[36] Tuxhorn JA Ayala GE Smith MJ Smith VC Dang TD Rowley DR . Reactive stroma in human prostate cancer: induction of myofibroblast phenotype and extracellular matrix remodeling. Clin Cancer Res. (2002) 8:2912–23. 12231536

[37] Burns-Cox N Avery NC Gingell JC Bailey AJ . Changes in collagen metabolism in prostate cancer: a host response that may alter progression. J Urol. (2001) 166:1698–701. doi: 10.1016/s0022-5347(05)65656-x. PMID: 11586205

[38] Koopmans G Hasse B Sinis N . Chapter 19: The role of collagen in peripheral nerve repair. Int Rev Neurobiol. (2009) 87:363–79. doi: 10.1016/s0074-7742(09)87019-0. PMID: 19682648

[39] Miao L Lin CM Huang L . Stromal barriers and strategies for the delivery of nanomedicine to desmoplastic tumors. J Control Release. (2015) 219:192–204. doi: 10.1016/j.jconrel.2015.08.017. PMID: 26277065 PMC4656082

[40] Brose K Bland KS Wang KH Arnott D Henzel W Goodman CS . Slit proteins bind Robo receptors and have an evolutionarily conserved role in repulsive axon guidance. Cell. (1999) 96:795–806. doi: 10.1016/s0092-8674(00)80590-5. PMID: 10102268

[41] Biankin AV Waddell N Kassahn KS Gingras MC Muthuswamy LB Johns AL . Pancreatic cancer genomes reveal aberrations in axon guidance pathway genes. Nature. (2012) 491:399–405. doi: 10.1038/nature11547. PMID: 23103869 PMC3530898

[42] Pinho AV van Bulck M Chantrill L Arshi M Sklyarova T Herrmann D . ROBO2 is a stroma suppressor gene in the pancreas and acts via TGF-β signalling. Nat Commun. (2018) 9:5083. doi: 10.1038/s41467-018-07497-z. PMID: 30504844 PMC6269509

[43] Padmanaban V Keller I Seltzer ES Ostendorf BN Kerner Z Tavazoie SF . Neuronal substance P drives metastasis through an extracellular RNA-TLR7 axis. Nature. (2024) 633:207–15. doi: 10.1038/s41586-024-07767-5. PMID: 39112700 PMC11633843

[44] Tavora B Mederer T Wessel KJ Ruffing S Sadjadi M Missmahl M . Tumoural activation of TLR3-SLIT2 axis in endothelium drives metastasis. Nature. (2020) 586:299–304. doi: 10.1038/s41586-020-2774-y. PMID: 32999457 PMC8088828

[45] Göhrig A Detjen KM Hilfenhaus G Körner JL Welzel M Arsenic R . Axon guidance factor SLIT2 inhibits neural invasion and metastasis in pancreatic cancer. Cancer Res. (2014) 74:1529–40. 10.1158/0008-5472.CAN-13-1012 24448236

[46] Lu G Du R Dong J Sun Y Zhou F Feng F . Cancer associated fibroblast derived SLIT2 drives gastric cancer cell metastasis by activating NEK9. Cell Death Dis. (2023) 14:421. doi: 10.1038/s41419-023-05965-z. PMID: 37443302 PMC10344862

[47] Francescone R Barbosa Vendramini-Costa D Franco-Barraza J Wagner J Muir A Lau AN . Netrin G1 promotes pancreatic tumorigenesis through cancer-associated fibroblast-driven nutritional support and immunosuppression. Cancer Discov. (2021) 11:446–79. doi: 10.1158/2159-8290.cd-20-0775. PMID: 33127842 PMC7858242

[48] Madeo M Colbert PL Vermeer DW Lucido CT Cain JT Vichaya EG . Cancer exosomes induce tumor innervation. Nat Commun. (2018) 9:4284. doi: 10.1038/s41467-018-06640-0. PMID: 30327461 PMC6191452

[49] Jessen KR Mirsky R . The origin and development of glial cells in peripheral nerves. Nat Rev Neurosci. (2005) 6:671–82. doi: 10.1038/nrn1746. PMID: 16136171

[50] Salzer J Feltri ML Jacob C . Schwann cell development and myelination. Cold Spring Harbor Perspect Biol. (2024) 16(9). doi: 10.1101/cshperspect.a020529. PMID: 38503507 PMC11368196

[51] Jessen KR Mirsky R Lloyd AC . Schwann cells: development and role in nerve repair. Cold Spring Harbor Perspect Biol. (2015) 7:a020487. doi: 10.1101/cshperspect.a020487. PMID: 25957303 PMC4484967

[52] Wekerle H Schwab M Linington C Meyermann R . Antigen presentation in the peripheral nervous system: Schwann cells present endogenous myelin autoantigens to lymphocytes. Eur J Immunol. (1986) 16:1551–7. doi: 10.1002/eji.1830161214. PMID: 2434335

[53] Colomar A Robitaille R . Glial modulation of synaptic transmission at the neuromuscular junction. Glia. (2004) 47:284–9. doi: 10.1002/glia.20086. PMID: 15252818

[54] Deborde S Omelchenko T Lyubchik A Zhou Y He S McNamara WF . Schwann cells induce cancer cell dispersion and invasion. J Clin Invest. (2016) 126:1538–54. doi: 10.1172/jci82658. PMID: 26999607 PMC4811155

[55] Demir IE Boldis A Pfitzinger PL Teller S Brunner E Klose N . Investigation of Schwann cells at neoplastic cell sites before the onset of cancer invasion. J Natl Cancer Inst. (2014) 106(8). doi: 10.1093/jnci/dju184. PMID: 25106646

[56] Shan C Wei J Hou R Wu B Yang Z Wang L . Schwann cells promote EMT and the Schwann-like differentiation of salivary adenoid cystic carcinoma cells via the BDNF/TrkB axis. Oncol Rep. (2016) 35:427–35. doi: 10.3892/or.2015.4366. PMID: 26530352

[57] Sroka IC Chopra H Das L Gard JM Nagle RB Cress AE . Schwann cells increase prostate and pancreatic tumor cell invasion using laminin binding A6 integrin. J Cell Biochem. (2016) 117:491–9. doi: 10.1002/jcb.25300. PMID: 26239765 PMC4809241

[58] Demir IE Friess H Ceyhan GO . Neural plasticity in pancreatitis and pancreatic cancer. Nat Rev Gastroenterol Hepatol. (2015) 12:649–59. doi: 10.1038/nrgastro.2015.166. PMID: 26460352

[59] Cravioto H . The role of Schwann cells in the development of human peripheral nerves. An electron microscopic study. J Ultrastruct Res. (1965) 12:634–51. doi: 10.1016/s0022-5320(65)80053-3. PMID: 5831054

[60] Zhang W He R Yang W Zhang Y Yuan Q Wang J . Autophagic Schwann cells promote perineural invasion mediated by the NGF/ATG7 paracrine pathway in pancreatic cancer. J Exp Clin Cancer Res: CR. (2022) 41:48. doi: 10.1186/s13046-021-02198-w. PMID: 35109895 PMC8809009

[61] Sakamoto Y Kitajima Y Edakuni G Sasatomi E Mori M Kitahara K . Expression of Trk tyrosine kinase receptor is a biologic marker for cell proliferation and perineural invasion of human pancreatic ductal adenocarcinoma. Oncol Rep. (2001) 8:477–84. doi: 10.3892/or.8.3.477. PMID: 11295066

[62] Ceyhan GO Bergmann F Kadihasanoglu M Erkan M Park W Hinz U . The neurotrophic factor artemin influences the extent of neural damage and growth in chronic pancreatitis. Gut. (2007) 56:534–44. doi: 10.1136/gut.2006.105528. PMID: 17047099 PMC1856869

[63] Bunge MB Wood PM Tynan LB Bates ML Sanes JR . Perineurium originates from fibroblasts: demonstration *in vitro* with a retroviral marker. Sci (New York NY). (1989) 243:229–31. doi: 10.1126/science.2492115. PMID: 2492115

[64] Neuberger TJ Cornbrooks CJ . Transient modulation of Schwann cell antigens after peripheral nerve transection and subsequent regeneration. J Neurocytology. (1989) 18:695–710. doi: 10.1007/bf01187088. PMID: 2515258

[65] Weitz J Garg B Martsinkovskiy A Patel S Tiriac H Lowy AM . Pancreatic ductal adenocarcinoma induces neural injury that promotes a transcriptomic and functional repair signature by peripheral neuroglia. Oncogene. (2023) 42:2536–46. doi: 10.1038/s41388-023-02775-7. PMID: 37433986 PMC10880465

[66] Deborde S Gusain L Powers A Marcadis A Yu Y Chen CH . Reprogrammed Schwann cells organize into dynamic tracks that promote pancreatic cancer invasion. Cancer Discov. (2022) 12:2454–73. doi: 10.1158/2159-8290.cd-21-1690. PMID: 35881881 PMC9533012

[67] Shurin GV Kruglov O Ding F Lin Y Hao X Keskinov AA . Melanoma-induced reprogramming of Schwann cell signaling aids tumor growth. Cancer Res. (2019) 79:2736–47. doi: 10.1158/0008-5472.can-18-3872. PMID: 30914431 PMC6522315

[68] Cao S Wang Y Zhou Y Zhang Y Ling X Zhang L . A novel therapeutic target for small-cell lung cancer: Tumor-associated repair-like Schwann cells. Cancers. (2022) 14(24). doi: 10.3390/cancers14246132. PMID: 36551618 PMC9776631

[69] Scheib J Höke A . Advances in peripheral nerve regeneration. Nat Rev Neurol. (2013) 9:668–76. doi: 10.1038/nrneurol.2013.227. PMID: 24217518

[70] Secq V Leca J Bressy C Guillaumond F Skrobuk P Nigri J . Stromal SLIT2 impacts on pancreatic cancer-associated neural remodeling. Cell Death Dis. (2015) 6:e1592. doi: 10.1038/cddis.2014.557. PMID: 25590802 PMC4669755

[71] Bressy C Lac S Nigri J Leca J Roques J Lavaut MN . LIF drives neural remodeling in pancreatic cancer and offers a new candidate biomarker. Cancer Res. (2018) 78:909–21. doi: 10.1158/0008-5472.can-15-2790. PMID: 29269518

[72] Xue ML Zhu YW Jiang YS Han LJ Shi MM Su R . Schwann cells regulate tumor cells and cancer-associated fibroblasts in the pancreatic ductal adenocarcinoma microenvironment. Nat Commun. (2023) 14(1). doi: 10.1038/s41467-023-40314-w. PMID: 37524695 PMC10390497

[73] García-Reyes B Kuzmanov I Schneider R Schneiker B Efferz P Kalff JC . Glial cell-derived soluble factors increase the metastatic potential of pancreatic adenocarcinoma cells and induce epithelial-to-mesenchymal transition. J Cancer Res Clin Oncol. (2023) 149:14315–27. doi: 10.1007/s00432-023-05133-y. PMID: 37572121 PMC10590291

[74] Han S Wang D Huang Y Zeng Z Xu P Xiong H . A reciprocal feedback between colon cancer cells and Schwann cells promotes the proliferation and metastasis of colon cancer. J Exp Clin Cancer Res. (2022) 41:348. doi: 10.1186/s13046-022-02556-2. PMID: 36522730 PMC9753336

[75] Gregory E Powers I Jamshidi-Parsian A Griffin R Song Y . Pancreatic tumor-derived extracellular vesicles stimulate Schwann cell phenotype indicative of perineural invasion via IL-8 signaling. bioRxiv. (2023).

[76] Guo Y Gil Z . The role of extracellular vesicles in cancer-nerve crosstalk of the peripheral nervous system. Cells. (2022) 11(8). doi: 10.3390/cells11081294. PMID: 35455973 PMC9027707

[77] Deuchars SA Lall VK . Sympathetic preganglionic neurons: properties and inputs. Compr Physiol. (2015) 5:829–69. doi: 10.1002/j.2040-4603.2015.tb00613.x. PMID: 25880515

[78] Sloan EK Priceman SJ Cox BF Yu S Pimentel MA Tangkanangnukul V . The sympathetic nervous system induces a metastatic switch in primary breast cancer. Cancer Res. (2010) 70:7042–52. doi: 10.1158/0008-5472.can-10-0522. PMID: 20823155 PMC2940980

[79] Thaker PH Han LY Kamat AA Arevalo JM Takahashi R Lu C . Chronic stress promotes tumor growth and angiogenesis in a mouse model of ovarian carcinoma. Nat Med. (2006) 12:939–44. doi: 10.1038/nm1447. PMID: 16862152

[80] Renz BW Tanaka T Sunagawa M Takahashi R Jiang Z Macchini M . Cholinergic signaling via muscarinic receptors directly and indirectly suppresses pancreatic tumorigenesis and cancer stemness. Cancer Discov. (2018) 8:1458–73. doi: 10.1158/2159-8290.cd-18-0046. PMID: 30185628 PMC6214763

[81] Renz BW Takahashi R Tanaka T Macchini M Hayakawa Y Dantes Z . β2 adrenergic-neurotrophin feedforward loop promotes pancreatic cancer. Cancer Cell. (2018) 33:75–90.e7. doi: 10.1016/j.ccell.2018.10.010. PMID: 29249692 PMC5760435

[82] Dallman MF . Complexities of stress. Sci (New York NY). (1992) 258:1970–1. doi: 10.1126/science.258.5090.1970. PMID: 17836191

[83] Li W Zhang J Gao Y Kong X Sun X . Nervous system in hepatocellular carcinoma: Correlation, mechanisms, therapeutic implications, and future perspectives. Biochim Biophys Acta Rev Cancer. (2025) 1880:189345. doi: 10.1016/j.bbcan.2025.189345. PMID: 40355012

[84] Kobayashi H Iida T Ochiai Y Malagola E Zhi X White RA . Neuro-mesenchymal interaction mediated by a β2-adrenergic nerve growth factor feedforward loop promotes colorectal cancer progression. Cancer Discov. (2025) 15:202–26. doi: 10.1158/2159-8290.cd-24-0287. PMID: 39137067 PMC11729495

[85] Ceyhan GO Demir IE Rauch U Bergmann F Müller MW Büchler MW . Pancreatic neuropathy results in "neural remodeling" and altered pancreatic innervation in chronic pancreatitis and pancreatic cancer. Am J Gastroenterol. (2009) 104:2555–65. doi: 10.1038/ajg.2009.380. PMID: 19568227

[86] Creed SJ Le CP Hassan M Pon CK Albold S Chan KT . β2-adrenoceptor signaling regulates invadopodia formation to enhance tumor cell invasion. Breast Cancer Res: BCR. (2015) 17:145. doi: 10.1186/s13058-015-0655-3. PMID: 26607426 PMC4660629

[87] Le CP Nowell CJ Kim-Fuchs C Botteri E Hiller JG Ismail H . Chronic stress in mice remodels lymph vasculature to promote tumour cell dissemination. Nat Commun. (2016) 7:10634. doi: 10.1038/ncomms10634. PMID: 26925549 PMC4773495

[88] Globig AM Zhao S Roginsky J Maltez VI Guiza J Avina-Ochoa N . The β(1)-adrenergic receptor links sympathetic nerves to T cell exhaustion. Nature. (2023) 622:383–92. 10.1038/s41586-023-06568-6 PMC10871066 37731001

[89] Guo K Ma Q Li J Wang Z Shan T Li W . Interaction of the sympathetic nerve with pancreatic cancer cells promotes perineural invasion through the activation of STAT3 signaling. Mol Cancer Ther. (2013) 12:264–73. doi: 10.1158/1535-7163.mct-12-0809. PMID: 23288783

[90] Xu Q Wang Z Chen X Duan W Lei J Zong L . Stromal-derived factor-1α/CXCL12-CXCR4 chemotactic pathway promotes perineural invasion in pancreatic cancer. Oncotarget. (2015) 6:4717–32. doi: 10.18632/oncotarget.3069. PMID: 25605248 PMC4467110

[91] Hanoun M Maryanovich M Arnal-Estapé A Frenette PS . Neural regulation of hematopoiesis, inflammation, and cancer. Neuron. (2015) 86:360–73. doi: 10.1016/j.neuron.2015.01.026. PMID: 25905810 PMC4416657

[92] Tomellini E Touil Y Lagadec C Julien S Ostyn P Ziental-Gelus N . Nerve growth factor and proNGF simultaneously promote symmetric self-renewal, quiescence, and epithelial to mesenchymal transition to enlarge the breast cancer stem cell compartment. Stem Cells (Dayton Ohio). (2015) 33:342–53. doi: 10.1002/stem.1849. PMID: 25286822

[93] Daquinag AC Gao Z Yu Y Kolonin MG . Endothelial TrkA coordinates vascularization and innervation in thermogenic adipose tissue and can be targeted to control metabolism. Mol Metab. (2022) 63:101544. doi: 10.2139/ssrn.3640842 35835372 PMC9310128

[94] Blaszkiewicz M Gunsch G Willows J Gardner M Sepeda J Sas A . Adipose tissue myeloid-lineage neuroimmune cells express genes important for neural plasticity and regulate adipose innervation. Front Endocrinol. (2022) 13:864925. doi: 10.3389/fendo.2022.864925. PMID: 35795142 PMC9251313

[95] Dobrenis K Gauthier LR Barroca V Magnon C . Granulocyte colony-stimulating factor off-target effect on nerve outgrowth promotes prostate cancer development. Int J Cancer. (2015) 136:982–8. doi: 10.1002/ijc.29046. PMID: 24975135

[96] Kannan Y Stead RH Goldsmith CH Bienenstock J . Lymphoid tissues induce NGF-dependent and NGF-independent neurite outgrowth from rat superior cervical ganglia explants in culture. J Neurosci Res. (1994) 37:374–83. doi: 10.1002/jnr.490370309. PMID: 8176759

[97] Barbany G Friedman WJ Persson H . Lymphocyte-mediated regulation of neurotransmitter gene expression in rat sympathetic ganglia. J Neuroimmunol. (1991) 32:97–104. doi: 10.1016/0165-5728(91)90001-n. PMID: 1672871

[98] Kannan Y Bienenstock J Ohta M Stanisz AM Stead RH . Nerve growth factor and cytokines mediate lymphoid tissue-induced neurite outgrowth from mouse superior cervical ganglia *in vitro*. J Immunol. (1996) 157:313–20. doi: 10.4049/jimmunol.157.1.313 8683132

[99] Straub RH Stebner K Härle P Kees F Falk W Schölmerich J . Key role of the sympathetic microenvironment for the interplay of tumour necrosis factor and interleukin 6 in normal but not in inflamed mouse colon mucosa. Gut. (2005) 54:1098–106. doi: 10.1136/gut.2004.062877. PMID: 15845563 PMC1774899

[100] Wang YN Tang Y He Z Ma H Wang L Liu Y . Slit3 secreted from M2-like macrophages increases sympathetic activity and thermogenesis in adipose tissue. Nat Metab. (2021) 3:1536–51. doi: 10.1038/s42255-021-00482-9. PMID: 34782792

[101] Pirzgalska RM Seixas E Seidman JS Link VM Sánchez NM Mahú I . Sympathetic neuron-associated macrophages contribute to obesity by importing and metabolizing norepinephrine. Nat Med. (2017) 23:1309–18. doi: 10.1038/nm.4422. PMID: 29035364 PMC7104364

[102] Rached MT Millership SJ Pedroni SMA Choudhury AI Costa ASH Hardy DG . Deletion of myeloid IRS2 enhances adipose tissue sympathetic nerve function and limits obesity. Mol Metab. (2019) 20:38–50. doi: 10.1016/j.molmet.2018.11.010. PMID: 30553769 PMC6358539

[103] Amit M Takahashi H Dragomir MP Lindemann A Gleber-Netto FO Pickering CR . Loss of p53 drives neuron reprogramming in head and neck cancer. Nature. (2020) 578:449–54. doi: 10.1038/s41586-020-1996-3. PMID: 32051587 PMC9723538

[104] Burnstock G . Autonomic neurotransmission: 60 years since sir Henry Dale. Annu Rev Pharmacol Toxicol. (2009) 49:1–30. doi: 10.1146/annurev.pharmtox.052808.102215. PMID: 18834312

[105] Vaes N Idris M Boesmans W Alves MM Melotte V . Nerves in gastrointestinal cancer: from mechanism to modulations. Nat Rev Gastroenterol Hepatol. (2022) 19:768–84. doi: 10.1038/s41575-022-00669-9. PMID: 36056202

[106] Hu J Chen W Shen L Chen Z Huang J . Crosstalk between the peripheral nervous system and breast cancer influences tumor progression. Biochim Biophys Acta Rev Cancer. (2022) 1877:188828. doi: 10.1016/j.bbcan.2022.188828. PMID: 36283598

[107] Zhou H Shi B Jia Y Qiu G Yang W Li J . Expression and significance of autonomic nerves and α9 nicotinic acetylcholine receptor in colorectal cancer. Mol Med Rep. (2018) 17:8423–31. doi: 10.3892/mmr.2018.8883. PMID: 29658602

[108] Zhang L Wu LL Huan HB Chen XJ Wen XD Yang DP . Sympathetic and parasympathetic innervation in hepatocellular carcinoma. Neoplasma. (2017) 64:840–6. doi: 10.4149/neo_2017_605. PMID: 28895408

[109] Yin QQ Xu LH Zhang M Xu C . Muscarinic acetylcholine receptor M1 mediates prostate cancer cell migration and invasion through hedgehog signaling. Asian J Androl. (2018) 20:608–14. doi: 10.4103/aja.aja_55_18. PMID: 30027929 PMC6219293

[110] Cheng K Shang AC Drachenberg CB Zhan M Raufman JP . Differential expression of M3 muscarinic receptors in progressive colon neoplasia and metastasis. Oncotarget. (2017) 8:21106–14. doi: 10.18632/oncotarget.15500. PMID: 28416748 PMC5400569

[111] Raufman JP Shant J Xie G Cheng K Gao XM Shiu B . Muscarinic receptor subtype-3 gene ablation and scopolamine butylbromide treatment attenuate small intestinal neoplasia in Apcmin/+ mice. Carcinogenesis. (2011) 32:1396–402. doi: 10.1093/carcin/bgr118. PMID: 21705482 PMC3165126

[112] Tolaymat M Larabee SM Hu S Xie G Raufman JP . The role of M3 muscarinic receptor ligand-induced kinase signaling in colon cancer progression. Cancers (Basel). (2019) 11(3). doi: 10.3390/cancers11030308. PMID: 30841571 PMC6468573

[113] Aronowitz AL Ali SR Glaun MDE Amit M . Acetylcholine in carcinogenesis and targeting cholinergic receptors in oncology. Adv Biol. (2022) 6:e2200053. doi: 10.1002/adbi.202200053. PMID: 35858206

[114] Sales ME Español AJ Salem AR Pulido PM Sanchez Y Sanchez F . Role of muscarinic acetylcholine receptors in breast cancer: Design of metronomic chemotherapy. Curr Clin Pharmacol. (2019) 14:91–100. doi: 10.2174/1574884714666181203095437. PMID: 30501602 PMC7011678

[115] Cavel O Shomron O Shabtay A Vital J Trejo-Leider L Weizman N . Endoneurial macrophages induce perineural invasion of pancreatic cancer cells by secretion of GDNF and activation of RET tyrosine kinase receptor. Cancer Res. (2012) 72:5733–43. doi: 10.1158/0008-5472.can-12-0764. PMID: 22971345

[116] Lee CH Huang CS Chen CS Tu SH Wang YJ Chang YJ . Overexpression and activation of the alpha9-nicotinic receptor during tumorigenesis in human breast epithelial cells. J Natl Cancer Institute. (2010) 102:1322–35. doi: 10.1093/jnci/djq300. PMID: 20733118

[117] Schuller HM . Is cancer triggered by altered signalling of nicotinic acetylcholine receptors? Nat Rev Cancer. (2009) 9:195–205. doi: 10.1038/nrc2590. PMID: 19194381

[118] Grando SA . Connections of nicotine to cancer. Nat Rev Cancer. (2014) 14:419–29. doi: 10.1038/nrc3725. PMID: 24827506

[119] Zhang L Guo L Tao M Fu W Xiu D . Parasympathetic neurogenesis is strongly associated with tumor budding and correlates with an adverse prognosis in pancreatic ductal adenocarcinoma. Chin J Cancer Res = Chung-Kuo Yen Cheng Yen Chiu. (2016) 28:180–86. doi: 10.21147/j.issn.1000-9604.2016.02.05. PMID: 27199515 PMC4865610

[120] Olofsson PS Rosas-Ballina M Levine YA Tracey KJ . Rethinking inflammation: neural circuits in the regulation of immunity. Immunol Rev. (2012) 248:188–204. doi: 10.1111/j.1600-065x.2012.01138.x. PMID: 22725962 PMC4536565

[121] Pavlov VA Tracey KJ . The vagus nerve and the inflammatory reflex--linking immunity and metabolism. Nat Rev Endocrinol. (2012) 8:743–54. doi: 10.1038/nrendo.2012.189. PMID: 23169440 PMC4082307

[122] Partecke LI Käding A Trung DN Diedrich S Sendler M Weiss F . Subdiaphragmatic vagotomy promotes tumor growth and reduces survival via TNFα in a murine pancreatic cancer model. Oncotarget. (2017) 8:22501–12. doi: 10.18632/oncotarget.15019. PMID: 28160574 PMC5410240

[123] Erin N Barkan GA Clawson GA . Vagus nerve regulates breast cancer metastasis to the adrenal gland. Anticancer Res. (2013) 33:3675–82. 24023295

[124] Yoshikawa H Kurokawa M Ozaki N Nara K Atou K Takada E . Nicotine inhibits the production of proinflammatory mediators in human monocytes by suppression of I-kappaB phosphorylation and nuclear factor-kappaB transcriptional activity through nicotinic acetylcholine receptor alpha7. Clin Exp Immunol. (2006) 146:116–23. doi: 10.1111/j.1365-2249.2006.03169.x. PMID: 16968406 PMC1809735

[125] Guarini S Altavilla D Cainazzo MM Giuliani D Bigiani A Marini H . Efferent vagal fibre stimulation blunts nuclear factor-kappaB activation and protects against hypovolemic hemorrhagic shock. Circulation. (2003) 107:1189–94. doi: 10.1161/01.cir.0000050627.90734.ed. PMID: 12615800

[126] Huston JM Ochani M Rosas-Ballina M Liao H Ochani K Pavlov VA . Splenectomy inactivates the cholinergic antiinflammatory pathway during lethal endotoxemia and polymicrobial sepsis. J Exp Med. (2006) 203:1623–28. doi: 10.1084/jem.20052362. PMID: 16785311 PMC2118357

[127] Egberts JH Cloosters V Noack A Schniewind B Thon L Klose S . Anti-tumor necrosis factor therapy inhibits pancreatic tumor growth and metastasis. Cancer Res. (2008) 68:1443–50. doi: 10.1158/0008-5472.can-07-5704. PMID: 18316608

[128] Antonica A Magni F Mearini L Paolocci N . Vagal control of lymphocyte release from rat thymus. J Auton Nerv Syst. (1994) 48:187–97. doi: 10.1016/0165-1838(94)90047-7. PMID: 7963254

[129] Mihaylova S Schweighöfer H Hackstein H Rosengarten B . Effects of anti-inflammatory vagus nerve stimulation in endotoxemic rats on blood and spleen lymphocyte subsets. Inflammation Res. (2014) 63:683–90. doi: 10.1007/s00011-014-0741-5. PMID: 24802890

[130] Lasser SA Ozbay Kurt FG Arkhypov I Utikal J Umansky V . Myeloid-derived suppressor cells in cancer and cancer therapy. Nat Rev Clin Oncol. (2024) 21:147–64. doi: 10.1038/s41571-023-00846-y. PMID: 38191922

[131] Zheng W Song H Luo Z Wu H Chen L Wang Y . Acetylcholine ameliorates colitis by promoting IL-10 secretion of monocytic myeloid-derived suppressor cells through the nAChR/ERK pathway. Proc Natl Acad Sci USA. (2021) 118(11). doi: 10.1073/pnas.2017762118. PMID: 33836585 PMC7980392

[132] Zhang Z Yu Q Zhang X Wang X Su Y He W . Electroacupuncture regulates inflammatory cytokines by activating the vagus nerve to enhance antitumor immunity in mice with breast tumors. Life Sci. (2021) 272:119259. doi: 10.1016/j.lfs.2021.119259. PMID: 33636172

[133] Sun P Zhou K Wang S Li P Chen S Lin G . Involvement of MAPK/NF-κB signaling in the activation of the cholinergic anti-inflammatory pathway in experimental colitis by chronic vagus nerve stimulation. PloS One. (2013) 8:e69424. doi: 10.1371/journal.pone.0069424. PMID: 23936328 PMC3732220

[134] Cox MA Bassi C Saunders ME Nechanitzky R Morgado-Palacin I Zheng C . Beyond neurotransmission: acetylcholine in immunity and inflammation. J Intern Med. (2020) 287:120–33. doi: 10.1111/joim.13006. PMID: 31710126

[135] Rosas-Ballina M Olofsson PS Ochani M Valdés-Ferrer SI Levine YA Reardon C . Acetylcholine-synthesizing T cells relay neural signals in a vagus nerve circuit. Science. (2011) 334:98–101. doi: 10.1126/science.1209985. PMID: 21921156 PMC4548937

[136] Dubeykovskaya Z Si Y Chen X Worthley DL Renz BW Urbanska AM . Neural innervation stimulates splenic TFF2 to arrest myeloid cell expansion and cancer. Nat Commun. (2016) 7:10517. doi: 10.1038/ncomms10517. PMID: 26841680 PMC4742920

[137] Craig AD . Pain mechanisms: labeled lines versus convergence in central processing. Annu Rev Neurosci. (2003) 26:1–30. doi: 10.1201/9781003567677-11 12651967

[138] Drossman DA . Chronic functional abdominal pain. Am J Gastroenterol. (1996) 91:2270–81. doi: 10.1007/s11938-000-0045-4. PMID: 8931402

[139] Di Sebastiano P Fink T Di Mola FF Weihe E Innocenti P Friess H . Neuroimmune appendicitis. Lancet (London England). (1999) 354:461–6. doi: 10.1016/s0140-6736(98)10463-4. PMID: 10465170

[140] Vera-Portocarrero LP Xie JY Kowal J Ossipov MH King T Porreca F . Descending facilitation from the rostral ventromedial medulla maintains visceral pain in rats with experimental pancreatitis. Gastroenterology. (2006) 130:2155–64. doi: 10.1053/j.gastro.2006.03.025. PMID: 16762636

[141] Schweizerhof M Stösser S Kurejova M Njoo C Gangadharan V Agarwal N . Hematopoietic colony-stimulating factors mediate tumor-nerve interactions and bone cancer pain. Nat Med. (2009) 15:802–7. doi: 10.1038/nm.1976. PMID: 19525966

[142] Selvaraj D Gangadharan V Michalski CW Kurejova M Stösser S Srivastava K . A functional role for VEGFR1 expressed in peripheral sensory neurons in cancer pain. Cancer Cell. (2015) 27:780–96. doi: 10.1016/j.ccell.2015.07.009. PMID: 26058077 PMC4469373

[143] Hirth M Gandla J Höper C Gaida MM Agarwal N Simonetti M . CXCL10 and CCL21 promote migration of pancreatic cancer cells toward sensory neurons and neural remodeling in tumors in mice, associated with pain in patients. Gastroenterology. (2020) 159:665–81.e13. doi: 10.1053/j.gastro.2020.04.037. PMID: 32330476

[144] Ceyhan GO Bergmann F Kadihasanoglu M Altintas B Demir IE Hinz U . Pancreatic neuropathy and neuropathic pain--a comprehensive pathomorphological study of 546 cases. Gastroenterology. (2009) 136:177–86.e1. doi: 10.1053/j.gastro.2008.09.029. PMID: 18992743

[145] Bockman DE Buchler M Malfertheiner P Beger HG . Analysis of nerves in chronic pancreatitis. Gastroenterology. (1988) 94:1459–69. doi: 10.1016/0016-5085(88)90687-7. PMID: 3360267

[146] Demir IE Ceyhan GO Rauch U Altintas B Klotz M Müller MW . The microenvironment in chronic pancreatitis and pancreatic cancer induces neuronal plasticity. Neurogastroenterol Motil. (2010) 22:480–90:e112-3. doi: 10.1111/j.1365-2982.2009.01428.x. PMID: 19912545

[147] Ceyhan GO Schäfer KH Kerscher AG Rauch U Demir IE Kadihasanoglu M . Nerve growth factor and artemin are paracrine mediators of pancreatic neuropathy in pancreatic adenocarcinoma. Ann Surg. (2010) 251:923–31. doi: 10.1097/sla.0b013e3181d974d4. PMID: 20395845

[148] Li Y Yu H Li ZM Yin KW Jin SY Chen CC . Colorectal cancer cells hijack a brain-gut polysynaptic circuit from the lateral septum to enteric neurons to sustain tumor growth. Nat Cancer. (2025). doi: 10.17504/protocols.io.bp2l68be5gqe/v2. PMID: 40841473

[149] Van Westerloo DJ Giebelen IA Florquin S Bruno MJ Larosa GJ Ulloa L . The vagus nerve and nicotinic receptors modulate experimental pancreatitis severity in mice. Gastroenterology. (2006) 130:1822–30. doi: 10.1053/j.gastro.2006.02.022. PMID: 16697744

[150] Lewin GR Barde YA . Physiology of the neurotrophins. Annu Rev Neurosci. (1996) 19:289–317. doi: 10.1146/annurev.neuro.19.1.289. PMID: 8833445

[151] Kaplan DR Hempstead BL Martin-Zanca D Chao MV Parada LF . The trk proto-oncogene product: a signal transducing receptor for nerve growth factor. Sci (New York NY). (1991) 252:554–8. doi: 10.1126/science.1850549. PMID: 1850549

[152] Klein R Jing SQ Nanduri V O'Rourke E Barbacid M . The trk proto-oncogene encodes a receptor for nerve growth factor. Cell. (1991) 65:189–97. doi: 10.1016/0092-8674(91)90419-y. PMID: 1849459

[153] Klein R Nanduri V Jing SA Lamballe F Tapley P Bryant S . The trkB tyrosine protein kinase is a receptor for brain-derived neurotrophic factor and neurotrophin-3. Cell. (1991) 66:395–403. doi: 10.1016/0092-8674(91)90628-c. PMID: 1649702 PMC2710095

[154] Squinto SP Stitt TN Aldrich TH Davis S Blanco SM Radziejewski C . trkB encodes a functional receptor for brain-derived neurotrophic factor and neurotrophin-3 but not nerve growth factor. Cell. (1991) 65:885–93. doi: 10.1016/0092-8674(91)90395-f. PMID: 1710174

[155] Klein R Lamballe F Bryant S Barbacid M . The trkB tyrosine protein kinase is a receptor for neurotrophin-4. Neuron. (1992) 8:947–56. doi: 10.1016/0896-6273(92)90209-v. PMID: 1375038

[156] Huang EJ Reichardt LF . Trk receptors: roles in neuronal signal transduction. Annu Rev Biochem. (2003) 72:609–42. doi: 10.1146/annurev.biochem.72.121801.161629. PMID: 12676795

[157] Johnson D Lanahan A Buck CR Sehgal A Morgan C Mercer E . Expression and structure of the human NGF receptor. Cell. (1986) 47:545–54. doi: 10.1016/0092-8674(86)90619-7. PMID: 3022937

[158] Rodriguez-Tébar A Dechant G Barde YA . Binding of brain-derived neurotrophic factor to the nerve growth factor receptor. Neuron. (1990) 4:487–92. doi: 10.1016/0896-6273(90)90107-q. PMID: 2157470

[159] Caporali A Meloni M Nailor A Mitić T Shantikumar S Riu F . p75(NTR)-dependent activation of NF-κB regulates microRNA-503 transcription and pericyte-endothelial crosstalk in diabetes after limb ischaemia. Nat Commun. (2015) 6:8024. doi: 10.1038/ncomms9024. PMID: 26268439 PMC4538859

[160] Riccio A Ahn S Davenport CM Blendy JA Ginty DD . Mediation by a CREB family transcription factor of NGF-dependent survival of sympathetic neurons. Sci (New York NY). (1999) 286:2358–61. doi: 10.1126/science.286.5448.2358. PMID: 10600750

[161] Freemont AJ Peacock TE Goupille P Hoyland JA O'Brien J Jayson MI . Nerve ingrowth into diseased intervertebral disc in chronic back pain. Lancet (London England). (1997) 350:178–81. doi: 10.1016/s0140-6736(97)02135-1. PMID: 9250186

[162] Harrington AW Ginty DD . Long-distance retrograde neurotrophic factor signalling in neurons. Nat Rev Neurosci. (2013) 14:177–87. doi: 10.1038/nrn3253. PMID: 23422909

[163] Levi-Montalcini R Aloe L Mugnaini E Oesch F Thoenen H . Nerve growth factor induces volume increase and enhances tyrosine hydroxylase synthesis in chemically axotomized sympathetic ganglia of newborn rats. PNAS. (1975) 72:595–99. doi: 10.1126/science.187.4172.113. PMID: 235759 PMC432360

[164] Lindsay RM Harmar AJ . Nerve growth factor regulates expression of neuropeptide genes in adult sensory neurons. Nature. (1989) 337:362–4. doi: 10.1038/337362a0. PMID: 2911387

[165] Leslie TA Emson PC Dowd PM Woolf CJ . Nerve growth factor contributes to the up-regulation of growth-associated protein 43 and preprotachykinin A messenger RNAs in primary sensory neurons following peripheral inflammation. Neuroscience. (1995) 67:753–61. doi: 10.1016/0306-4522(95)00101-n. PMID: 7675201

[166] Levi-Montalcini R . The nerve growth factor: thirty-five years later. EMBO J. (1987) 6:1145–54. doi: 10.1007/bf01116861. PMID: 3301324 PMC553912

[167] Chao MV Bothwell MA Ross AH Koprowski H Lanahan AA Buck CR . Gene transfer and molecular cloning of the human NGF receptor. Sci (New York NY). (1986) 232:518–21. doi: 10.1126/science.3008331. PMID: 3008331

[168] Radeke MJ Misko TP Hsu C Herzenberg LA Shooter EM . Gene transfer and molecular cloning of the rat nerve growth factor receptor. Nature. (1987) 325:593–7. doi: 10.1038/325593a0. PMID: 3027580

[169] Nykjaer A Willnow TE . Sortilin: a receptor to regulate neuronal viability and function. Trends Neurosci. (2012) 35:261–70. doi: 10.1016/j.tins.2012.01.003. PMID: 22341525

[170] Marsland M Dowdell A Jiang CC Wilmott JS Scolyer RA Zhang XD . Expression of NGF/proNGF and their receptors trkA, p75(NTR) and sortilin in melanoma. Int J Mol Sci. (2022) 23(8). doi: 10.3390/ijms23084260. PMID: 35457078 PMC9032112

[171] Gschwind A Fischer OM Ullrich A . The discovery of receptor tyrosine kinases: targets for cancer therapy. Nat Rev Cancer. (2004) 4:361–70. doi: 10.1038/nrc1360. PMID: 15122207

[172] Marchetti D Aucoin R Blust J Murry B Greiter-Wilke A . p75 neurotrophin receptor functions as a survival receptor in brain-metastatic melanoma cells. J Cell Biochem. (2004) 91:206–15. doi: 10.1002/jcb.10649. PMID: 14689592

[173] Khwaja F Allen J Lynch J Andrews P Djakiew D . Ibuprofen inhibits survival of bladder cancer cells by induced expression of the p75NTR tumor suppressor protein. Cancer Res. (2004) 64:6207–13. doi: 10.1158/0008-5472.can-03-3814. PMID: 15342406

[174] Jin H Pan Y He L Zhai H Li X Zhao L . p75 neurotrophin receptor inhibits invasion and metastasis of gastric cancer. Mol Cancer Res: MCR. (2007) 5:423–33. doi: 10.1158/1541-7786.mcr-06-0407. PMID: 17510309

[175] Conroy JN Coulson EJ . High-affinity TrkA and p75 neurotrophin receptor complexes: A twisted affair. J Biol Chem. (2022) 298:101568. doi: 10.1016/j.jbc.2022.101568. PMID: 35051416 PMC8889134

[176] Yoon SO Casaccia-Bonnefil P Carter B Chao MV . Competitive signaling between TrkA and p75 nerve growth factor receptors determines cell survival. J Neurosci. (1998) 18:3273–81. doi: 10.1523/jneurosci.18-09-03273.1998. PMID: 9547236 PMC6792655

[177] Lagadec C Meignan S Adriaenssens E Foveau B Vanhecke E Romon R . TrkA overexpression enhances growth and metastasis of breast cancer cells. Oncogene. (2009) 28:1960–70. doi: 10.1038/onc.2009.61. PMID: 19330021

[178] Descamps S Pawlowski V Révillion F Hornez L Hebbar M Boilly B . Expression of nerve growth factor receptors and their prognostic value in human breast cancer. Cancer Res. (2001) 61:4337–40. 11389056

[179] Jung HH Kim JY Cho EY Oh JM Lee JE Kim SW . Elevated level of nerve growth factor (NGF) in serum-derived exosomes predicts poor survival in patients with breast cancer undergoing neoadjuvant chemotherapy. Cancers. (2021) 13(21). doi: 10.3390/cancers13215260. PMID: 34771423 PMC8582365

[180] Dollé L El Yazidi-Belkoura I Adriaenssens E Nurcombe V Hondermarck H . Nerve growth factor overexpression and autocrine loop in breast cancer cells. Oncogene. (2003) 22:5592–601. 10.1038/sj.onc.1206805 12944907

[181] Descamps S Lebourhis X Delehedde M Boilly B Hondermarck H . Nerve growth factor is mitogenic for cancerous but not normal human breast epithelial cells. J Biol Chem. (1998) 273:16659–62. doi: 10.1074/jbc.273.27.16659. PMID: 9642218

[182] Descamps S Toillon RA Adriaenssens E Pawlowski V Cool SM Nurcombe V . Nerve growth factor stimulates proliferation and survival of human breast cancer cells through two distinct signaling pathways. J Biol Chem. (2001) 276:17864–70. doi: 10.1074/jbc.m010499200. PMID: 11359788

[183] Di Donato M Galasso G Giovannelli P Sinisi AA Migliaccio A Castoria G . Targeting the nerve growth factor signaling impairs the proliferative and migratory phenotype of triple-negative breast cancer cells. Front Cell Dev Biol. (2021) 9:676568. doi: 10.3389/fcell.2021.676568. PMID: 34268306 PMC8275826

[184] Demont Y Corbet C Page A Ataman-Önal Y Choquet-Kastylevsky G Fliniaux I . Pro-nerve growth factor induces autocrine stimulation of breast cancer cell invasion through tropomyosin-related kinase A (TrkA) and sortilin protein. J Biol Chem. (2012) 287:1923–31. doi: 10.1074/jbc.m110.211714. PMID: 22128158 PMC3265873

[185] Lévêque R Corbet C Aubert L Guilbert M Lagadec C Adriaenssens E . ProNGF increases breast tumor aggressiveness through functional association of TrkA with EphA2. Cancer Lett. (2019) 449:196–206. 30771434 10.1016/j.canlet.2019.02.019

[186] Romon R Adriaenssens E Lagadec C Germain E Hondermarck H Le Bourhis X . Nerve growth factor promotes breast cancer angiogenesis by activating multiple pathways. Mol Cancer. (2010) 9:157. doi: 10.1186/1476-4598-9-157. PMID: 20569463 PMC2901260

[187] Vermeulen L De Sousa E Melo F Richel DJ Medema JP . The developing cancer stem-cell model: clinical challenges and opportunities. Lancet Oncol. (2012) 13:e83–9. doi: 10.1016/s1470-2045(11)70257-1. PMID: 22300863

[188] Zhang Y Li W Guo S Wu Z Zhang L Liu Y . FBXO22 mediates the NGF/TRKA signaling pathway in bone metastases in prostate cancer. Am J Pathol. (2023) 193:1248–66. doi: 10.1016/j.ajpath.2023.05.012. PMID: 37301536

[189] Camilleri-Broët S Cremer I Marmey B Comperat E Viguié F Audouin J . TRAF4 overexpression is a common characteristic of human carcinomas. Oncogene. (2007) 26:142–7. doi: 10.1038/sj.onc.1209762. PMID: 16799635

[190] Singh R Karri D Shen H Shao J Dasgupta S Huang S . TRAF4-mediated ubiquitination of NGF receptor TrkA regulates prostate cancer metastasis. J Clin Invest. (2018) 128:3129–43. doi: 10.1172/jci96060. PMID: 29715200 PMC6026011

[191] Di Donato M Cernera G Migliaccio A Castoria G . Nerve growth factor induces proliferation and aggressiveness in prostate cancer cells. Cancers. (2019) 11(6). doi: 10.3390/cancers11060784. PMID: 31174415 PMC6627659

[192] Anagnostopoulou V Pediaditakis I Alkahtani S Alarifi SA Schmidt EM Lang F . Differential effects of dehydroepiandrosterone and testosterone in prostate and colon cancer cell apoptosis: the role of nerve growth factor (NGF) receptors. Endocrinology. (2013) 154:2446–56. doi: 10.1210/en.2012-2249. PMID: 23696568

[193] Lazaridis I Charalampopoulos I Alexaki VI Avlonitis N Pediaditakis I Efstathopoulos P . Neurosteroid dehydroepiandrosterone interacts with nerve growth factor (NGF) receptors, preventing neuronal apoptosis. PloS Biol. (2011) 9:e1001051. doi: 10.1371/journal.pbio.1001051. PMID: 21541365 PMC3082517

[194] Goda M Atagi S Amitani K Hobara N Kitamura Y Kawasaki H . Nerve growth factor suppresses prostate tumor growth. J Pharmacol Sci. (2010) 112:463–6. doi: 10.1254/jphs.09354sc. PMID: 20308799

[195] Krygier S Djakiew D . Neurotrophin receptor p75(NTR) suppresses growth and nerve growth factor-mediated metastasis of human prostate cancer cells. Int J Cancer. (2002) 98:1–7. doi: 10.1002/ijc.10160. PMID: 11857376

[196] Pflug BR Onoda M Lynch JH Djakiew D . Reduced expression of the low affinity nerve growth factor receptor in benign and Malignant human prostate tissue and loss of expression in four human metastatic prostate tumor cell lines. Cancer Res. (1992) 52:5403–6. doi: 10.1210/endo.136.1.7828539. PMID: 1382843

[197] Peng T Guo Y Gan Z Ling Y Xiong J Liang X . Nerve growth factor (NGF) encourages the neuroinvasive potential of pancreatic cancer cells by activating the Warburg effect and promoting tumor derived exosomal miRNA-21 expression. Oxid Med Cell Longevity. (2022) 2022:8445093. doi: 10.1155/2022/8445093. PMID: 36285300 PMC9588358

[198] Liebl F Demir IE Rosenberg R Boldis A Yildiz E Kujundzic K . The severity of neural invasion is associated with shortened survival in colon cancer. Clin Cancer Res. (2013) 19:50–61. doi: 10.1158/1078-0432.ccr-12-2392. PMID: 23147996

[199] Bapat AA Munoz RM Von Hoff DD Han H . Blocking nerve growth factor signaling reduces the neural invasion potential of pancreatic cancer cells. PloS One. (2016) 11:e0165586. doi: 10.1371/journal.pone.0165586. PMID: 27792755 PMC5085053

[200] Qin T Xiao Y Qian W Wang X Gong M Wang Q . HGF/c-Met pathway facilitates the perineural invasion of pancreatic cancer by activating the mTOR/NGF axis. Cell Death Dis. (2022) 13:387. doi: 10.1038/s41419-022-04799-5. PMID: 35449152 PMC9023560

[201] Du JJ Dou KF Peng SY Qian BZ Xiao HS Liu F . Expression of NGF family and their receptors in gastric carcinoma: a cDNA microarray study. World J Gastroenterol. (2003) 9:1431–4. doi: 10.3748/wjg.v9.i7.1431. PMID: 12854135 PMC4615477

[202] Dou N Yang D Yu S Wu B Gao Y Li Y . SNRPA enhances tumour cell growth in gastric cancer through modulating NGF expression. Cell Proliferation. (2018) 51:e12484. doi: 10.1111/cpr.12484. PMID: 30039889 PMC6528855

[203] Du H Rosbash M . The U1 snRNP protein U1C recognizes the 5' splice site in the absence of base pairing. Nature. (2002) 419:86–90. doi: 10.1038/nature00947. PMID: 12214237

[204] Ho R Eggert A Hishiki T Minturn JE Ikegaki N Foster P . Resistance to chemotherapy mediated by TrkB in neuroblastomas. Cancer Res. (2002) 62:6462–6. 12438236

[205] Pearse RN Swendeman SL Li Y Rafii D Hempstead BL . A neurotrophin axis in myeloma: TrkB and BDNF promote tumor-cell survival. Blood. (2005) 105:4429–36. doi: 10.1182/blood-2004-08-3096. PMID: 15657181

[206] Jaboin J Kim CJ Kaplan DR Thiele CJ . Brain-derived neurotrophic factor activation of TrkB protects neuroblastoma cells from chemotherapy-induced apoptosis via phosphatidylinositol 3'-kinase pathway. Cancer Res. (2002) 62:6756–63. 12438277

[207] Douma S van Laar T Zevenhoven J Meuwissen R Van Garderen E Peeper DS . Suppression of anoikis and induction of metastasis by the neurotrophic receptor TrkB. Nature. (2004) 430:1034–9. doi: 10.1038/nature02765. PMID: 15329723

[208] Guo D Hou X Zhang H Sun W Zhu L Liang J . More expressions of BDNF and TrkB in multiple hepatocellular carcinoma and anti-BDNF or K252a induced apoptosis, supressed invasion of HepG2 and HCCLM3 cells. J Exp Clin Cancer Res: CR. (2011) 30:97. doi: 10.1186/1756-9966-30-97. PMID: 21999199 PMC3212909

[209] Lam CT Yang ZF Lau CK Tam KH Fan ST Poon RT . Brain-derived neurotrophic factor promotes tumorigenesis via induction of neovascularization: implication in hepatocellular carcinoma. Clin Cancer Res. (2011) 17:3123–33. doi: 10.1158/1078-0432.ccr-10-2802. PMID: 21421859

[210] Matsumoto K Wada RK Yamashiro JM Kaplan DR Thiele CJ . Expression of brain-derived neurotrophic factor and p145TrkB affects survival, differentiation, and invasiveness of human neuroblastoma cells. Cancer Res. (1995) 55:1798–806. 7712490

[211] Martens LK Kirschner KM Warnecke C Scholz H . Hypoxia-inducible factor-1 (HIF-1) is a transcriptional activator of the TrkB neurotrophin receptor gene. J Biol Chem. (2007) 282:14379–88. doi: 10.1074/jbc.m609857200. PMID: 17374610

[212] Eggert A Grotzer MA Ikegaki N Liu XG Evans AE Brodeur GM . Expression of the neurotrophin receptor TrkA down-regulates expression and function of angiogenic stimulators in SH-SY5Y neuroblastoma cells. Cancer Res. (2002) 62:1802–8. 11912158

[213] Nakamura K Martin KC Jackson JK Beppu K Woo CW Thiele CJ . Brain-derived neurotrophic factor activation of TrkB induces vascular endothelial growth factor expression via hypoxia-inducible factor-1alpha in neuroblastoma cells. Cancer Res. (2006) 66:4249–55. doi: 10.1158/0008-5472.can-05-2789. PMID: 16618748

[214] Lucarelli E Kaplan D Thiele CJ . Activation of trk-A but not trk-B signal transduction pathway inhibits growth of neuroblastoma cells. Eur J Cancer (Oxford England: 1990). (1997) 33:2068–70. doi: 10.1016/s0959-8049(97)00266-9. PMID: 9516854

[215] Kermani P Rafii D Jin DK Whitlock P Schaeffer W Chiang A . Neurotrophins promote revascularization by local recruitment of TrkB+ endothelial cells and systemic mobilization of hematopoietic progenitors. J Clin Invest. (2005) 115:653–63. doi: 10.1172/jci200522655. PMID: 15765148 PMC1051987

[216] Brodeur GM . Neuroblastoma: biological insights into a clinical enigma. Nat Rev Cancer. (2003) 3:203–16. doi: 10.1038/nrc1014. PMID: 12612655

[217] Yang J Siao CJ Nagappan G Marinic T Jing D McGrath K . Neuronal release of proBDNF. Nat Neurosci. (2009) 12:113–5. doi: 10.1038/nn.2244. PMID: 19136973 PMC2737352

[218] Xiong J Zhou L Yang M Lim Y Zhu YH Fu DL . ProBDNF and its receptors are upregulated in glioma and inhibit the growth of glioma cells *in vitro*. Neuro-Oncology. (2013) 15:990–1007. doi: 10.1093/neuonc/not039. PMID: 23576602 PMC3714150

[219] Xiong J Zhou L Lim Y Yang M Zhu YH Li ZW . Mature BDNF promotes the growth of glioma cells *in vitro*. Oncol Rep. (2013) 30:2719–24. doi: 10.3892/or.2013.2746. PMID: 24064679

[220] Sinkevicius KW Kriegel C Bellaria KJ Lee J Lau AN Leeman KT . Neurotrophin receptor TrkB promotes lung adenocarcinoma metastasis. PNAS. (2014) 111:10299–304. doi: 10.1073/pnas.1404399111. PMID: 24982195 PMC4104911

[221] Kupferman ME Jiffar T El-Naggar A Yilmaz T Zhou G Xie T . TrkB induces EMT and has a key role in invasion of head and neck squamous cell carcinoma. Oncogene. (2010) 29:2047–59. doi: 10.1038/onc.2009.486. PMID: 20101235 PMC3138334

[222] Okugawa Y Tanaka K Inoue Y Kawamura M Kawamoto A Hiro J . Brain-derived neurotrophic factor/tropomyosin-related kinase B pathway in gastric cancer. Br J Cancer. (2013) 108:121–30. doi: 10.1038/bjc.2012.499. PMID: 23175149 PMC3553513

[223] Zhang Y Fujiwara Y Doki Y Takiguchi S Yasuda T Miyata H . Overexpression of tyrosine kinase B protein as a predictor for distant metastases and prognosis in gastric carcinoma. Oncology. (2008) 75:17–26. doi: 10.1159/000151615. PMID: 18719350

[224] Choi B Lee EJ Shin MK Park YS Ryu MH Kim SM . Upregulation of brain-derived neurotrophic factor in advanced gastric cancer contributes to bone metastatic osteolysis by inducing long pentraxin 3. Oncotarget. (2016) 7:55506–17. doi: 10.18632/oncotarget.10747. PMID: 27458153 PMC5342432

[225] Mazouffre C Geyl S Perraud A Blondy S Jauberteau MO Mathonnet M . Dual inhibition of BDNF/TrkB and autophagy: a promising therapeutic approach for colorectal cancer. J Cell Mol Med. (2017) 21:2610–22. doi: 10.1111/jcmm.13181. PMID: 28597984 PMC5618676

[226] Tanaka K Okugawa Y Toiyama Y Inoue Y Saigusa S Kawamura M . Brain-derived neurotrophic factor (BDNF)-induced tropomyosin-related kinase B (Trk B) signaling is a potential therapeutic target for peritoneal carcinomatosis arising from colorectal cancer. PloS One. (2014) 9:e96410. doi: 10.1371/journal.pone.0096410. PMID: 24801982 PMC4011754

[227] Sasahira T Ueda N Kurihara M Matsushima S Ohmori H Fujii K . Tropomyosin receptor kinases B and C are tumor progressive and metastatic marker in colorectal carcinoma. Hum Pathol. (2013) 44:1098–106. doi: 10.1016/j.humpath.2012.09.016. PMID: 23332094

[228] Smit MA Geiger TR Song JY Gitelman I Peeper DS . A Twist-Snail axis critical for TrkB-induced epithelial-mesenchymal transition-like transformation, anoikis resistance, and metastasis. Mol Cell Biol. (2009) 29:3722–37. doi: 10.1128/mcb.01164-08. PMID: 19414595 PMC2698746

[229] Fujikawa H Tanaka K Toiyama Y Saigusa S Inoue Y Uchida K . High TrkB expression levels are associated with poor prognosis and EMT induction in colorectal cancer cells. J Gastroenterol. (2012) 47:775–84. doi: 10.1007/s00535-012-0532-0. PMID: 22361863

[230] Montagut C Dalmases A Bellosillo B Crespo M Pairet S Iglesias M . Identification of a mutation in the extracellular domain of the Epidermal Growth Factor Receptor conferring cetuximab resistance in colorectal cancer. Nat Med. (2012) 18:221–3. doi: 10.1038/nm.2609. PMID: 22270724

[231] Diaz LA Williams RT Wu J Kinde I Hecht JR Berlin J . The molecular evolution of acquired resistance to targeted EGFR blockade in colorectal cancers. Nature. (2012) 486:537–40. doi: 10.1038/nature11219. PMID: 22722843 PMC3436069

[232] de Farias CB Heinen TE dos Santos RP Abujamra AL Schwartsmann G Roesler R . BDNF/TrkB signaling protects HT-29 human colon cancer cells from EGFR inhibition. Biochem Biophys Res Commun. (2012) 425:328–32. doi: 10.1016/j.bbrc.2012.07.091. PMID: 22842573

[233] Sclabas GM Fujioka S Schmidt C Li Z Frederick WA Yang W . Overexpression of tropomysin-related kinase B in metastatic human pancreatic cancer cells. Clin Cancer Res. (2005) 11:440–9. doi: 10.1158/1078-0432.440.11.2. PMID: 15701826

[234] Vanhecke E Adriaenssens E Verbeke S Meignan S Germain E Berteaux N . Brain-derived neurotrophic factor and neurotrophin-4/5 are expressed in breast cancer and can be targeted to inhibit tumor cell survival. Clin Cancer Res. (2011) 17:1741–52. doi: 10.1158/1078-0432.ccr-10-1890. PMID: 21350004

[235] Lamouille S Xu J Derynck R . Molecular mechanisms of epithelial-mesenchymal transition. Nat Rev Mol Cell Biol. (2014) 15:178–96. doi: 10.1038/nrm3758. PMID: 24556840 PMC4240281

[236] Howe EN Cochrane DR Richer JK . Targets of miR-200c mediate suppression of cell motility and anoikis resistance. Breast Cancer Res: BCR. (2011) 13:R45. doi: 10.1186/bcr2867. PMID: 21501518 PMC3219208

[237] Yin B Ma ZY Zhou ZW Gao WC Du ZG Zhao ZH . The TrkB+ cancer stem cells contribute to post-chemotherapy recurrence of triple-negative breast cancers in an orthotopic mouse model. Oncogene. (2015) 34:761–70. doi: 10.1038/onc.2014.8. PMID: 24531713

[238] Jia S Wang W Hu Z Shan C Wang L Wu B . BDNF mediated TrkB activation contributes to the EMT progression and the poor prognosis in human salivary adenoid cystic carcinoma. Oral Oncol. (2015) 51:64–70. doi: 10.1016/j.oraloncology.2014.10.008. PMID: 25456007

[239] Van Roy F . Beyond E-cadherin: roles of other cadherin superfamily members in cancer. Nat Rev Cancer. (2014) 14:121–34. doi: 10.1038/nrc3647. PMID: 24442140

[240] Kim MS Lee WS Jin W . TrkB promotes breast cancer metastasis via suppression of Runx3 and Keap1 expression. Mol Cells. (2016) 39:258–65. doi: 10.14348/molcells.2016.2310. PMID: 26657794 PMC4794608

[241] Innominato PF Libbrecht L van den Oord JJ . Expression of neurotrophins and their receptors in pigment cell lesions of the skin. J Pathol. (2001) 194:95–100. doi: 10.1002/path.861. PMID: 11329147

[242] Denkins Y Reiland J Roy M Sinnappah-Kang ND Galjour J Murry BP . Brain metastases in melanoma: roles of neurotrophins. Neuro-Oncology. (2004) 6:154–65. doi: 10.1215/s115285170300067x. PMID: 15134630 PMC1871977

[243] Marchetti D McQuillan DJ Spohn WC Carson DD Nicolson GL . Neurotrophin stimulation of human melanoma cell invasion: selected enhancement of heparanase activity and heparanase degradation of specific heparan sulfate subpopulations. Cancer Res. (1996) 56:2856–63. doi: 10.1016/s0065-2571(96)00019-2. PMID: 8665526

[244] Louie E Chen XF Coomes A Ji K Tsirka S Chen EI . Neurotrophin-3 modulates breast cancer cells and the microenvironment to promote the growth of breast cancer brain metastasis. Oncogene. (2013) 32:4064–77. doi: 10.1038/onc.2012.417. PMID: 23001042 PMC3998718

[245] Luo Y Kaz AM Kanngurn S Welsch P Morris SM Wang J . NTRK3 is a potential tumor suppressor gene commonly inactivated by epigenetic mechanisms in colorectal cancer. PloS Genet. (2013) 9:e1003552. doi: 10.1371/journal.pgen.1003552. PMID: 23874207 PMC3708790

[246] Genevois AL Ichim G Coissieux MM Lambert MP Lavial F Goldschneider D . Dependence receptor TrkC is a putative colon cancer tumor suppressor. PNAS. (2013) 110:3017–22. doi: 10.1073/pnas.1212333110. PMID: 23341610 PMC3581924

[247] Wang X Yan Y Li L Li T Thakur A Zhang K . The influence of neuro-tumor interactions on tumorigenesis and therapeutic response. Exp Hematol Oncol. (2026) 15:18. doi: 10.1186/s40164-026-00752-w. PMID: 41645346 PMC12882464

[248] Zhang Y Liao Q Wen X Fan J Yuan T Tong X . Hijacking of the nervous system in cancer: mechanism and therapeutic targets. Mol Cancer. (2025) 24:44. doi: 10.1186/s12943-025-02246-5. PMID: 39915765 PMC11800603

[249] Zhang L Zhu D Wang J Guo J Jiang M Qi X . The neuro-immune axis in cancer: mechanisms of innervation-driven tumor progression and therapeutic opportunities. Front Immunol. (2025) 16:1693419. doi: 10.3389/fimmu.2025.1693419. PMID: 41601691 PMC12833073

[250] Shi DD Guo JA Hoffman HI Su J Mino-Kenudson M Barth JL . Therapeutic avenues for cancer neuroscience: translational frontiers and clinical opportunities. Lancet Oncol. (2022) 23:e62–74. doi: 10.1016/s1470-2045(21)00596-9. PMID: 35114133 PMC9516432

[251] Park H Poo MM . Neurotrophin regulation of neural circuit development and function. Nat Rev Neurosci. (2013) 14:7–23. doi: 10.1038/nrn3379. PMID: 23254191

[252] Hutchings C Phillips JA Djamgoz MBA . Nerve input to tumours: Pathophysiological consequences of a dynamic relationship. Biochim Biophys Acta Rev Cancer. (2020) 1874:188411. doi: 10.1016/j.bbcan.2020.188411. PMID: 32828885

[253] Wang H Yu M Ochani M Amella CA Tanovic M Susarla S . Nicotinic acetylcholine receptor alpha7 subunit is an essential regulator of inflammation. Nature. (2003) 421:384–8. doi: 10.1038/nature01339. PMID: 12508119

[254] Coarfa C Florentin D Putluri N Ding Y Au J He D . Influence of the neural microenvironment on prostate cancer. Prostate. (2018) 78:128–39. doi: 10.1002/pros.23454. PMID: 29131367 PMC5836952

[255] Griffin N Faulkner S Jobling P Hondermarck H . Targeting neurotrophin signaling in cancer: The renaissance. Pharmacol Res. (2018) 135:12–7. doi: 10.1016/j.phrs.2018.07.019. PMID: 30031169

[256] Lei Y Tang L Xie Y Xianyu Y Zhang L Wang P . Gold nanoclusters-assisted delivery of NGF siRNA for effective treatment of pancreatic cancer. Nat Commun. (2017) 8:15130. doi: 10.1038/ncomms15130. PMID: 28440296 PMC5414062

[257] Liu X Hu Y Yu J Xue Y He X Jiang F . PLA-THF-PEG nanoparticles co-encapsulating AV3 and KH3 for synergistic pancreatic cancer therapy via stromal remodeling and metabolic inhibition. Front Pharmacol. (2026) 17:1723694. doi: 10.3389/fphar.2026.1723694. PMID: 41716313 PMC12913430

[258] Zuo S Wang Z Jiang X Zhao Y Wen P Wang J . Regulating tumor innervation by nanodrugs potentiates cancer immunochemotherapy and relieve chemotherapy-induced neuropathic pain. Biomaterials. (2024) 309:122603. doi: 10.1016/j.biomaterials.2024.122603. PMID: 38713972

[259] Pacenta HL Macy ME . Entrectinib and other ALK/TRK inhibitors for the treatment of neuroblastoma. Drug Des Dev Ther. (2018) 12:3549–61. doi: 10.2147/dddt.s147384. PMID: 30425456 PMC6204873

[260] Scott LJ . Larotrectinib: First global approval. Drugs. (2019) 79:201–6. doi: 10.1007/s40265-018-1044-x. PMID: 30635837

[261] Drilon A Laetsch TW Kummar S Dubois SG Lassen UN Demetri GD . Efficacy of larotrectinib in TRK fusion-positive cancers in adults and children. N Engl J Med. (2018) 378:731–9. doi: 10.1056/nejmoa1714448. PMID: 29466156 PMC5857389

[262] Wang T Zhang K Zhou Y . Interrogating physiological functions with light and chemicals. Annu Rev Physiol. (2026) 88:21–46. doi: 10.1146/annurev-physiol-042924-083733. PMID: 41212991 PMC13152683

[263] Thiel V Sur D Picoli CC McErlain T Couto K Simon DJ . Next-gen tools in cancer neuroscience. Cell Rep. (2025) 44:116258. doi: 10.1016/j.celrep.2025.116258. PMID: 40934086

[264] Gazova S Klena L Galvankova K Babula P Krizanova O . Role of adrenergic receptors and their blocking in cancer research. BioMed Pharmacother. (2025) 192:118637. doi: 10.1016/j.biopha.2025.118637. PMID: 41067075

[265] Cole SW Sood AK . Molecular pathways: Beta-adrenergic signaling in cancer. Clin Cancer Res. (2012) 18:1201–6. doi: 10.1158/1078-0432.ccr-11-0641. PMID: 22186256 PMC3294063

[266] Hiller JG Cole SW Crone EM Byrne DJ Shackleford DM Pang JB . Preoperative β-blockade with propranolol reduces biomarkers of metastasis in breast cancer: A phase II randomized trial. Clin Cancer Res. (2020) 26:1803–11. doi: 10.1158/1078-0432.ccr-19-2641. PMID: 31754048

[267] Zheng B Du P Zeng Z Cao P Ma X Jiang Y . Propranolol inhibits EMT and metastasis in breast cancer through miR-499-5p-mediated Sox6. J Cancer Res Clin Oncol. (2024) 150:59. doi: 10.1007/s00432-023-05599-w. PMID: 38294713 PMC10830604

[268] Liao P Song K Zhu Z Liu Z Zhang W Li W . Propranolol suppresses the growth of colorectal cancer through simultaneously activating autologous CD8(+) T cells and inhibiting tumor AKT/MAPK pathway. Clin Pharmacol Ther. (2020) 108:606–15. doi: 10.1002/cpt.1894. PMID: 32418204

[269] Powe DG Entschladen F . Targeted therapies: Using β-blockers to inhibit breast cancer progression. Nat Rev Clin Oncol. (2011) 8:511–2. doi: 10.1038/nrclinonc.2011.123. PMID: 21808268

[270] Grytli HH Fagerland MW Fosså SD Taskén KA . Association between use of β-blockers and prostate cancer-specific survival: A cohort study of 3561 prostate cancer patients with high-risk or metastatic disease. Eur Urol. (2014) 65:635–41. doi: 10.1016/j.eururo.2013.01.007. PMID: 23351721

[271] Barron TI Connolly RM Sharp L Bennett K Visvanathan K . Beta blockers and breast cancer mortality: A population- based study. J Clin Oncol. (2011) 29:2635–44. doi: 10.1200/jco.2010.33.5422. PMID: 21632503

[272] Melhem-Bertrandt A Chavez-Macgregor M Lei X Brown EN Lee RT Meric-Bernstam F . Beta-blocker use is associated with improved relapse-free survival in patients with triple-negative breast cancer. J Clin Oncol. (2011) 29:2645–52. doi: 10.1200/jco.2010.33.4441. PMID: 21632501 PMC3139371

[273] De Giorgi V Grazzini M Gandini S Benemei S Lotti T Marchionni N . Treatment with β-blockers and reduced disease progression in patients with thick melanoma. Arch Internal Med. (2011) 171:779–81. doi: 10.1001/archinternmed.2011.131. PMID: 21518948

[274] Lemeshow S Sørensen HT Phillips G Yang EV Antonsen S Riis AH . β-blockers and survival among Danish patients with Malignant melanoma: A population-based cohort study. Cancer Epidemiol Biomarkers Prev. (2011) 20:2273–9. doi: 10.1158/1055-9965.epi-11-0249. PMID: 21933972 PMC3652234

[275] De Giorgi V Gandini S Grazzini M Benemei S Marchionni N Geppetti P . Effect of β-blockers and other antihypertensive drugs on the risk of melanoma recurrence and death. Mayo Clinic Proc. (2013) 88:1196–203. doi: 10.1016/j.mayocp.2013.09.001. PMID: 24182700

[276] Udumyan R Montgomery S Fang F Almroth H Valdimarsdottir UA Ekbom A . Beta-blocker drug use and survival among patients with pancreatic adenocarcinoma. Cancer Res. (2017) 77:3700–7. doi: 10.1158/0008-5472.can-17-0108. PMID: 28473530

[277] Kanda H Ishii K Ogura Y Imamura T Kanai M Arima K . Naftopidil, a selective alpha-1 adrenoceptor antagonist, inhibits growth of human prostate cancer cells by G1 cell cycle arrest. Int J Cancer. (2008) 122:444–51. doi: 10.1002/ijc.23095. PMID: 17918159

[278] Hori Y Ishii K Kanda H Iwamoto Y Nishikawa K Soga N . Naftopidil, a selective {alpha}1-adrenoceptor antagonist, suppresses human prostate tumor growth by altering interactions between tumor cells and stroma. Cancer Prev Res (Philadelphia Pa). (2011) 4:87–96. doi: 10.1158/1940-6207.capr-10-0189. PMID: 21205739

[279] Ishii K Matsuoka I Kajiwara S Sasaki T Miki M Kato M . Additive naftopidil treatment synergizes docetaxel-induced apoptosis in human prostate cancer cells. J Cancer Res Clin Oncol. (2018) 144:89–98. doi: 10.1007/s00432-017-2536-x. PMID: 29098395 PMC11813469

[280] Iwamoto Y Ishii K Sasaki T Kato M Kanda H Yamada Y . Oral naftopidil suppresses human renal-cell carcinoma by inducing G(1) cell-cycle arrest in tumor and vascular endothelial cells. Cancer Prev Res (Philadelphia Pa). (2013) 6:1000–6. doi: 10.1158/1940-6207.capr-13-0095. PMID: 23901046

[281] Tsumura H Satoh T Ishiyama H Tabata K Kotani S Minamida S . Comparison of prophylactic naftopidil, tamsulosin, and silodosin for 125I brachytherapy-induced lower urinary tract symptoms in patients with prostate cancer: Randomized controlled trial. Int J Radiat Oncol Biol Phys. (2011) 81:e385–92. doi: 10.1016/j.ijrobp.2011.04.026. PMID: 21664068

[282] Mikami K Nagaya H Gotoh A Kanno T Tsuchiya A Nakano T . Naftopidil is useful for the treatment of Malignant pleural mesothelioma. Pharmacology. (2014) 94:163–9. doi: 10.1159/000368050. PMID: 25301502

[283] Lin XB Jiang L Ding MH Chen ZH Bao Y Chen Y . Anti-tumor activity of phenoxybenzamine hydrochloride on Malignant glioma cells. Tumour Biol. (2016) 37:2901–8. doi: 10.1007/s13277-015-4102-y. PMID: 26409450

[284] Jin MZ Jin WL . The updated landscape of tumor microenvironment and drug repurposing. Signal Transduction Targeted Ther. (2020) 5:166. doi: 10.1038/s41392-020-00280-x. PMID: 32843638 PMC7447642

[285] Xu R Shang C Zhao J Han Y Liu J Chen K . Activation of M3 muscarinic receptor by acetylcholine promotes non-small cell lung cancer cell proliferation and invasion via EGFR/PI3K/AKT pathway. Tumour Biol. (2015) 36:4091–100. doi: 10.1007/s13277-014-2911-z. PMID: 25964092

[286] Kodaira M Kajimura M Takeuchi K Lin S Hanai H Kaneko E . Functional muscarinic m3 receptor expressed in gastric cancer cells stimulates tyrosine phosphorylation and MAP kinase. J Gastroenterol. (1999) 34:163–71. doi: 10.1007/s005350050238. PMID: 10213113

[287] Nagy D Kosztka L Pap P Nagy Z Rusznák Z Csernoch L . Cytoplasmic Ca2+ concentration changes evoked by muscarinic cholinergic stimulation in primary and metastatic melanoma cell lines. Melanoma Res. (2011) 21:12–23. doi: 10.1097/cmr.0b013e3283414477. PMID: 21102359

[288] Sammi SR Rawat JK Raghav N Kumar A Roy S Singh M . Galantamine attenuates N,N-dimethyl hydrazine induced neoplastic colon damage by inhibiting acetylcholinesterase and bimodal regulation of nicotinic cholinergic neurotransmission. Eur J Pharmacol. (2018) 818:174–83. doi: 10.1016/j.ejphar.2017.10.036. PMID: 29074413

[289] Bernik TR Friedman SG Ochani M Diraimo R Ulloa L Yang H . Pharmacological stimulation of the cholinergic antiinflammatory pathway. J Exp Med. (2002) 195:781–8. doi: 10.1084/jem.20011714. PMID: 11901203 PMC2193742

[290] Erin N Duymuş O Oztürk S Demir N . Activation of vagus nerve by semapimod alters substance P levels and decreases breast cancer metastasis. Regul Pept. (2012) 179:101–8. doi: 10.1016/j.regpep.2012.08.001. PMID: 22982142

[291] Chang YC Liu CL Chen MJ Hsu YW Chen SN Lin CH . Local anesthetics induce apoptosis in human breast tumor cells. Anesth Analg. (2014) 118:116–24. doi: 10.1213/ane.0b013e3182a94479. PMID: 24247230

[292] Galoş EV Tat TF Popa R Efrimescu CI Finnerty D Buggy DJ . Neutrophil extracellular trapping and angiogenesis biomarkers after intravenous or inhalation anaesthesia with or without intravenous lidocaine for breast cancer surgery: A prospective, randomised trial. Br J Anaesth. (2020) 125:712–21. 10.1016/j.bja.2020.05.003 32616309

[293] Idevall-Hagren O Dickson EJ Hille B Toomre DK De Camilli P . Optogenetic control of phosphoinositide metabolism. PNAS. (2012) 109:E2316–23. doi: 10.1073/pnas.1211305109. PMID: 22847441 PMC3435206

[294] Wang Y Buck A Piel B Zerefa L Murugan N Coherd CD . Affinity fine-tuning anti-CAIX CAR-T cells mitigate on-target off-tumor side effects. Mol Cancer. (2024) 23:56. doi: 10.1186/s12943-024-01952-w. PMID: 38491381 PMC10943873

[295] Cattaruzza F Nazeer A To M Hammond M Koski C Liu LY . Precision-activated T-cell engagers targeting HER2 or EGFR and CD3 mitigate on-target, off-tumor toxicity for immunotherapy in solid tumors. Nat Cancer. (2023) 4:485–501. doi: 10.1038/s43018-023-00536-9. PMID: 36997747 PMC10132983

[296] Nguyen NT Huang K Zeng H Jing J Wang R Fang S . Nano-optogenetic engineering of CAR T cells for precision immunotherapy with enhanced safety. Nat Nanotechnol. (2021) 16:1424–34. doi: 10.1038/s41565-021-00982-5. PMID: 34697491 PMC8678207

[297] Schiller M Azulay-Debby H Boshnak N Elyahu Y Korin B Ben-Shaanan TL . Optogenetic activation of local colonic sympathetic innervations attenuates colitis by limiting immune cell extravasation. Immunity. (2021) 54:1022–1036.e8. doi: 10.1016/j.immuni.2021.04.007. PMID: 33932356 PMC8116309

[298] Wrighton NC Farrell FX Chang R Kashyap AK Barbone FP Mulcahy LS . Small peptides as potent mimetics of the protein hormone erythropoietin. Sci (New York NY). (1996) 273:458–64. doi: 10.1126/science.273.5274.458. PMID: 8662529

[299] Dooley CT Chung NN Wilkes BC Schiller PW Bidlack JM Pasternak GW . An all D-amino acid opioid peptide with central analgesic activity from a combinatorial library. Sci (New York NY). (1994) 266:2019–22. doi: 10.1126/science.7801131. PMID: 7801131

[300] Longo FM Massa SM . Small-molecule modulation of neurotrophin receptors: A strategy for the treatment of neurological disease. Nat Rev Drug Discov. (2013) 12:507–25. doi: 10.1038/nrd4024. PMID: 23977697

[301] Denk F Bennett DL Mcmahon SB . Nerve growth factor and pain mechanisms. Annu Rev Neurosci. (2017) 40:307–25. doi: 10.1146/annurev-neuro-072116-031121. PMID: 28441116

[302] Brasca MG Amboldi N Ballinari D Cameron A Casale E Cervi G . Identification of N,1,4,4-tetramethyl-8-{[4-(4-methylpiperazin-1-yl)phenyl]amino}-4,5-dihydro-1H-pyrazolo[4,3-h]quinazoline-3-carboxamide (PHA-848125), a potent, orally available cyclin dependent kinase inhibitor. J Med Chem. (2009) 52:5152–63. doi: 10.1021/jm9006559. PMID: 19603809

[303] Albanese C Alzani R Amboldi N Degrassi A Festuccia C Fiorentini F . Anti-tumour efficacy on glioma models of PHA-848125, a multi-kinase inhibitor able to cross the blood-brain barrier. Br J Pharmacol. (2013) 169:156–66. doi: 10.1158/1535-7163.targ-09-pr-3. PMID: 23347136 PMC3632246

[304] Buehlmann D Ielacqua GD Xandry J Rudin M . Prospective administration of anti-nerve growth factor treatment effectively suppresses functional connectivity alterations after cancer-induced bone pain in mice. Pain. (2019) 160:151–9. doi: 10.1097/j.pain.0000000000001388. PMID: 30161041

[305] Pezet S Mcmahon SB . Neurotrophins: mediators and modulators of pain. Annu Rev Neurosci. (2006) 29:507–38. doi: 10.1146/annurev.neuro.29.051605.112929. PMID: 16776595

[306] Fallon M Sopata M Dragon E Brown MT Viktrup L West CR . A randomized placebo-controlled trial of the anti-nerve growth factor antibody tanezumab in subjects with cancer pain due to bone metastasis. Oncologist. (2023) 28:e1268–78. doi: 10.1093/oncolo/oyad188. PMID: 37343145 PMC10712717

[307] Jimenez-Andrade JM Ghilardi JR Castañeda-Corral G Kuskowski MA Mantyh PW . Preventive or late administration of anti-NGF therapy attenuates tumor-induced nerve sprouting, neuroma formation, and cancer pain. Pain. (2011) 152:2564–74. doi: 10.1016/j.pain.2011.07.020. PMID: 21907491 PMC3199350

[308] Mccaffrey G Thompson ML Majuta L Fealk MN Chartier S Longo G . NGF blockade at early times during bone cancer development attenuates bone destruction and increases limb use. Cancer Res. (2014) 74:7014–23. doi: 10.1158/0008-5472.can-14-1220. PMID: 25287160 PMC4253026

[309] Ugolini G Marinelli S Covaceuszach S Cattaneo A Pavone F . The function neutralizing anti-TrkA antibody MNAC13 reduces inflammatory and neuropathic pain. Proc Natl Acad Sci USA. (2007) 104:2985–90. doi: 10.1073/pnas.0611253104. PMID: 17301229 PMC1815293

[310] Smith M De Bono J Sternberg C Le Moulec S Oudard S De Giorgi U . Phase III study of cabozantinib in previously treated metastatic castration-resistant prostate cancer: COMET-1. J Clin Oncol. (2016) 34:3005–13. doi: 10.1200/jco.2015.65.5597. PMID: 27400947

[311] Shabbir M Stuart R . Lestaurtinib, a multitargeted tyrosine kinase inhibitor: from bench to bedside. Expert Opin Invest Drugs. (2010) 19:427–36. doi: 10.1517/13543781003598862. PMID: 20141349

[312] Ghilardi JR Freeman KT Jimenez-Andrade JM Mantyh WG Bloom A Bouhana KS . Sustained blockade of neurotrophin receptors TrkA, TrkB and TrkC reduces non-malignant skeletal pain but not the maintenance of sensory and sympathetic nerve fibers. Bone. (2011) 48:389–98. doi: 10.1016/j.bone.2010.09.019. PMID: 20854944 PMC3020250

[313] Baruch EN Gleber-Netto FO Nagarajan P Rao X Akhter S Eichwald T . Cancer-induced nerve injury promotes resistance to anti-PD-1 therapy. Nature. (2025). doi: 10.1038/s41586-025-09370-8. PMID: 40836096 PMC12406299

[314] Gandhi S Pandey MR Attwood K Ji W Witkiewicz AK Knudsen ES . Phase I clinical trial of combination propranolol and pembrolizumab in locally advanced and metastatic melanoma: Safety, tolerability, and preliminary evidence of antitumor activity. Clin Cancer Res. (2021) 27:87–95. doi: 10.1158/1078-0432.ccr-20-2381. PMID: 33127652 PMC7785669

[315] Kennedy OJ Kicinski M Valpione S Gandini S Suciu S Blank CU . Prognostic and predictive value of β-blockers in the EORTC 1325/KEYNOTE-054 phase III trial of pembrolizumab versus placebo in resected high-risk stage III melanoma. Eur J Cancer (Oxford England: 1990). (2022) 165:97–112. doi: 10.1016/j.ejca.2022.01.017. PMID: 35220182

[316] Fjæstad KY Rømer AMA Goitea V Johansen AZ Thorseth ML Carretta M . Blockade of beta-adrenergic receptors reduces cancer growth and enhances the response to anti-CTLA4 therapy by modulating the tumor microenvironment. Oncogene. (2022) 41:1364–75. 10.1038/s41388-021-02170-0 PMC8881216 35017664

[317] Kokolus KM Zhang Y Sivik JM Schmeck C Zhu J Repasky EA . Beta blocker use correlates with better overall survival in metastatic melanoma patients and improves the efficacy of immunotherapies in mice. Oncoimmunology. (2018) 7:e1405205. doi: 10.1080/2162402x.2017.1405205. PMID: 29399407 PMC5790362

[318] Ajmal I Farooq MA Duan Y Yao J Gao Y Hui X . Intrinsic ADRB2 inhibition improves CAR-T cell therapy efficacy against prostate cancer. Mol Ther. (2024) 32:3539–57. doi: 10.1016/j.ymthe.2024.08.028. PMID: 39228124 PMC11489547

[319] He H Guo J Hu Y Zhang H Li X Zhang J . Saikosaponin D reverses epinephrine- and norepinephrine-induced gemcitabine resistance in intrahepatic cholangiocarcinoma by downregulating ADRB2/glycolysis signaling. Acta Biochim Biophys Sin. (2023) 55:1404–14. doi: 10.3724/abbs.2023040. PMID: 37489008 PMC10520481

[320] Tjioe KC Cardoso DM Oliveira SHP Bernabe DG . Stress hormone norepinephrine incites resistance of oral cancer cells to chemotherapy. Endocrine-Related Cancer. (2022) 29:201–12. doi: 10.1530/erc-20-0460. PMID: 35099408

[321] Chang A Botteri E Gillis RD Lofling L Le CP Ziegler AI . Beta-blockade enhances anthracycline control of metastasis in triple-negative breast cancer. Sci Transl Med. (2023) 15:eadf1147. doi: 10.1126/scitranslmed.adf1147. PMID: 37099632

[322] Puzderova B Belvoncikova P Grossmannova K Csaderova L Labudova M Fecikova S . Propranolol, promising chemosensitizer and candidate for the combined therapy through disruption of tumor microenvironment homeostasis by decreasing the level of carbonic anhydrase IX. Int J Mol Sci. (2023) 24(13). doi: 10.3390/ijms241311094. PMID: 37446271 PMC10341831

[323] Lin Y Liu Y Gao Z Jing D Bi R Cui X . Beta-adrenergic receptor blocker propranolol triggers anti-tumor immunity and enhances irinotecan therapy in mice colorectal cancer. Eur J Pharmacol. (2023) 949:175718. doi: 10.1016/j.ejphar.2023.175718. PMID: 37054937

[324] Shan T Ma Q Zhang D Guo K Liu H Wang F . β2-adrenoceptor blocker synergizes with gemcitabine to inhibit the proliferation of pancreatic cancer cells via apoptosis induction. Eur J Pharmacol. (2011) 665:1–7. doi: 10.1016/j.ejphar.2011.04.055. PMID: 21570961

[325] Kim YJ Jang SK Kim G Hong SE Park CS Seong MK . Nebivolol sensitizes BT-474 breast cancer cells to FGFR inhibitors. Anticancer Res. (2023) 43:1973–80. doi: 10.21873/anticanres.16357. PMID: 37097659

[326] Florent R Weiswald LB Lambert B Brotin E Abeilard E Louis MH . Bim, Puma and Noxa upregulation by naftopidil sensitizes ovarian cancer to the BH3-mimetic ABT-737 and the MEK inhibitor trametinib. Cell Death Dis. (2020) 11:380. doi: 10.1038/s41419-020-2588-8. PMID: 32424251 PMC7235085

[327] Hanahan D Weinberg RA . Hallmarks of cancer: the next generation. Cell. (2011) 144:646–74. doi: 10.1016/j.cell.2011.02.013. PMID: 21376230

[328] Hanahan D Weinberg RA . The hallmarks of cancer. Cell. (2000) 100:57–70. doi: 10.1093/med/9780199656103.003.0001. PMID: 10647931

[329] Mancusi R Monje M . The neuroscience of cancer. Nature. (2023) 618:467–79. doi: 10.1038/s41586-023-05968-y. PMID: 37316719 PMC11146751

